# Paleoenvironment reconstruction and peat-forming conditions of Neogene paralic coal sequences from Mukah, Sarawak, Malaysia

**DOI:** 10.1038/s41598-022-12668-6

**Published:** 2022-05-25

**Authors:** Nor Syazwani Zainal Abidin, Khairul Azlan Mustapha, Wan Hasiah Abdullah, Zainey Konjing

**Affiliations:** 1 Department of Geology, Faculty of Science, University of Malaya, 50603 Kuala Lumpur, Malaysia; 2 Geosciences Department, Faculty of Science and Information Technology, Universiti Teknologi PETRONAS, 32610 Bandar Seri Iskandar, Perak Malaysia; 3 Southeast Asia Clastic and Carbonate Research Laboratory (SEACARL), Institute of Hydrocarbon Recovery for Enhanced Oil Recovery, Universiti Teknologi PETRONAS, 32610 Bandar Seri Iskandar, Perak Malaysia; 4 Geological Society of Malaysia, c/o Department of Geology, University of Malaya, 50603 Kuala Lumpur, Malaysia; 5 Orogenic Sdn. Bhd., Lot 6744, Jalan Tekali 1, Kawasan Perindustrian Tekali 1 1/2 Miles, Sungai Tekali, 43100 Hulu Langat, Selangor Malaysia

**Keywords:** Geochemistry, Petrology, Sedimentology

## Abstract

Eight coal seams containing Neogene paralic coals from the Mukah coalfield, Sarawak, Malaysia, were investigated using petrographical, palynological, and organic geochemical analyses to describe coal-forming vegetation, paleoclimatic, and paleoenvironment conditions during peat development and precursor mires, as well as their associations within a sequence-stratigraphic context. The petrographic and geochemical data of the coals imply the existence of oxygen-deficient and water-saturated conditions in the precursor mires. The reducing conditions in the mires were followed by biomass loss. The Mukah coals are suggested to be deposited in freshwater peat swamps, and the rich preservation of angiosperm pollen indicates that the organic matter in dense and lowland forest vegetation was mostly terrigenous. The overwhelming presence of *Casuarina* and *Calamus* types suggest the paleomires were closely linked to the Kerapah/Kerangas peat forest and were marginally bordered by rattan, which was supported by biomarker data. Rheotrophic–ombrotrophic mires temporarily formed due to water table fluctuations, which were strongly dependent on ever-wet climate changes and syn-depositional tectonics during the Neogene, and resulted in the balanced to high peat accumulation and preservation. A maximum thickness of 35 m of peat deposits is suggested to form between 10,000 and 175,000 years ago based on the peat:coal ratio. The coals are proposed to be influenced by transgressive to initial highstand cycles within the paralic setting.

## Introduction

**Figure 1:**
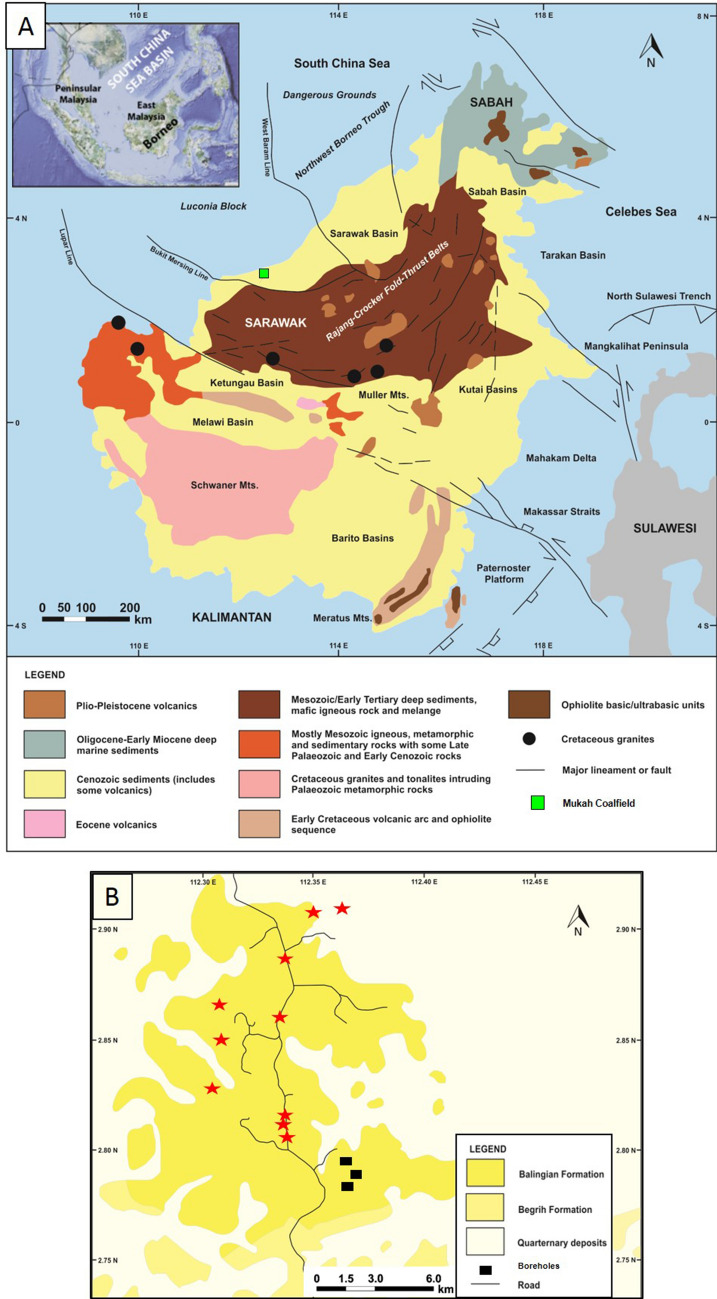
(**A**) Geological map of Borneo (modified from Widodo et al.^3^). (**B**) Location map of the Mukah coalfield and borehole location of this study. Note the red star symbols represent the sampling locations taken by Sia and Abdullah^6^, Sia et al.^1^, Hakimi et al.^9^, and Murtaza et al.^8^.

Coal deposits from the Mukah coalfield in Sarawak is of interest in this study as they are considered as one of the most prolific producers of humic coals in Malaysia^1^. Located between N02.78000° latitude and E112.37129° longitude (Fig. 1a, b), the coal was deposited during Early Miocene to Middle Miocene^1, 2^ and belongs to the Neogene coal-bearing sequence^3, 4^ of the Balingian Formation.

Earlier studies on the Mukah coals have emphasized the quality and re-estimation of coal reserves^1, 5–9^. However, the primary dataset used in paleogeographic evolution and clastic sedimentology of the coal-bearing strata (e.g.^7–9^) only concerns the significance of the whole coal seam as a single lithofacies within several depositional cycles. Therefore, there exists uncertainty due to the variety in type, setting, and maturity of the coal seams related to the cyclic nature of coal sequences as intra-seam variations tend to be related to the stacking or architecture of the benches inside the coal bed or seam, and may originate from different types of paleomires^10^. As the Mukah area is dominated by paralic coal, the coal succession is interpreted to be deposited in a near-coastal setting, which is more strongly affected by sea-level changes compared to inland deposits that are not hydrologically connected to the sea. In parallel with recent work by^11^, this study focuses on the high-resolution macroscopic and microscopic analyses integrated with organic geochemical data of the Balingian Formation, providing a bigger picture of the peat-to-coal evolution.

The main objective of this study is to provide significant insights into how the composition and stratigraphy of coal at a single locality change over time in response to changes in accommodation space, compaction, paleoclimate, and/or tectonic subsidence, allowing for the prediction of coal quality and its corresponding relation to the basis of sequence stratigraphy study of the Neogene paralic Mukah coal seams, using petrographical, geochemical, and palynological studies. The study mainly emphasizes intra-seams and whole seam variations of coals, and their associated sedimentary rocks.

### Geological setting

Borneo Island is located in East Malaysia, shared by the Malaysian states of Sarawak and Sabah, Kalimantan in Indonesia, and Brunei (Fig. 1a). The island is a product of multiple extensive tectonic events including the accretion of ophiolites as well as the formation of island arcs and microcontinental fragments during the Mesozoic^12–18^. In the Cenozoic, the geology of Borneo was formed primarily by the collision between the continental crusts of Luconia Block and the Sarawak margin during the Late Eocene, followed by collision between the Dangerous Grounds and NW Sabah during the late Early Miocene^14, 18^. This former Late Eocene tectonic collision formed a major unconformity on onshore central Sarawak known as the Sarawak Orogeny; whereas, the latter late Early Miocene tectonic collision subsequently resulted in the ‘Deep Regional Unconformity’ in offshore NW Sabah and the Sabah Orogeny onshore western Sabah^2, 18–20^. Furthermore, a major accretionary wedge complex was formed during the Late Cretaceous to Early Miocene, producing the Rajang-Crocker Fold-Thrust Belts and a peripheral foreland basin that extends from Sarawak to Sabah^2, 21, 22^ (Fig. 1a). The fold and thrust belts are also referred to as the Sibu Zone in onshore Sarawak^4, 7, 23^.

## Results

### Coal petrography

**Figure 2:**
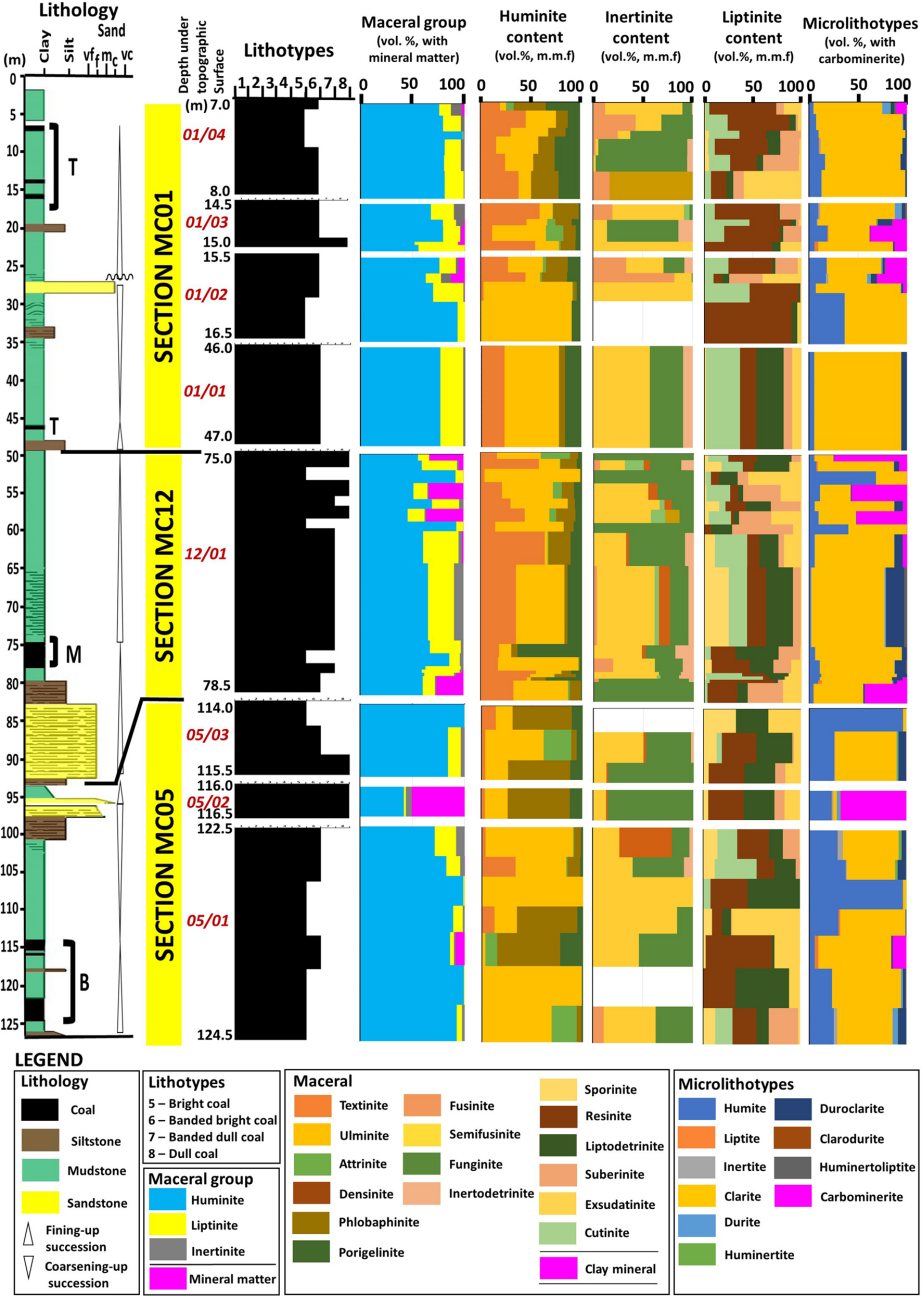
Complete vertical profile of the eight coal seams from Mukah coalfield shows upward compositional changes in lithotypes, maceral groups, macerals, mineral matter, and microlithotypes. Note coal seam is labeled as 01/04.

**Figure 3:**
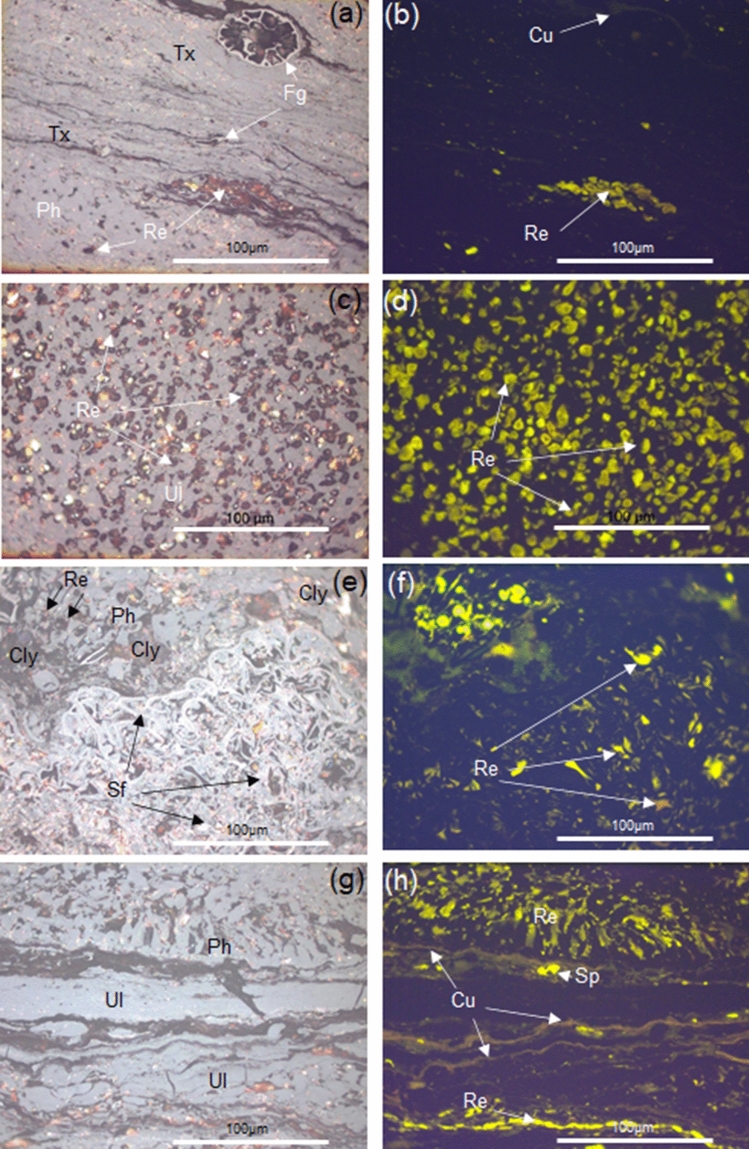
Photomicrographs of organic matter assemblages from the studied Mukah coals in the Balingian Formation; under white light (left) and reflected light (right): (**a**–**h**) resinite (Re), cutinite (Cu), sporinite (Sp), semifusinite (Sf), and funginite (Fg) associated with huminite macerals of textinite (Tx), ulminite (Ul) and phlobaphinite (Ph) as a dominant maceral group assemblage. Clay (Cly) matrix can be observed in (**e**,**f**).

Results from the petrographic studies of the studied coals are reported in Table 1 and the detailed compositional variations of the intra-seams within vertical profiles of the coal-bearing succession are illustrated in Figs. 2 and 3. The Mukah coals were dominated (average vol%) by the huminite group (41.9–91.9%) and mainly consisted of humotelinite which is subdivided into textinite (1.5–26.2%) and ulminite (9.5–55.1%). The composition of liptinite does not vary significantly with an average of 2.0–25.3% for all samples. Minor amounts of inertinite (1.4–5.5%) were found and characterized by fusinite, semifusinite, funginite, and inertodetrinite. Mineral matter mainly occurred as clay minerals and was typically dispersed. High amounts of argillaceous mineral matter (up to 50.6%) were reported in certain intra-seams. The amount of pyrite was almost non-existent and rarely found in most of the samples, therefore was not recorded in this study. The inertinite–huminite/vitrinite factor calculated was low for all samples, ranging from 2.0 to 11.7. In terms of microlithotypes, the Mukah coals were primarily characterized by clarite, humite, and duroclarite with an average of 3.5–88.7%, 5.5–61.1%, and 0.0–8.5%, respectively. Liptite, inertite, durite, huminertite, and huminertoliptite were discovered infrequently (< 1%). Carbominerite was observed in certain intra-seams depending on the mineral matter occurrences.

**Table 1:** The petrographic composition shows values in range and average (avg.) as total (vol%), and mineral-free basis (vol%, m.m.f.)/carbominerite free basis (vol%, c.m.f) of the intra-seams in the Mukah coals.

Coal section	MC 01						MC 12						MC 05						Avg	Avg
Seams	01/04	01/02–01/03	01/01	01/04	01/02–01/03	01/01	T	M	B	T	M	B	05/03	05/02	05/01	05/03	05/02	05/01		
Maceral	(vol%)			(vol%, mmf)			(vol%)			(vol%, mmf)			(vol%)			(vol%, mmf)			(vol%)	(vol%, mmf)
Textinite	12.8–46.7	0.0–39.8	17.5	12.8–46.7	0.0–39.8	17.5	3.8–34.3	13.6–38.7	12.4–34.4	3.8–36.0	13.6–39.3	12.4–34.4	3.2–14.1	1.5	0.0–27.4	3.2–14.1	3.1	0.0–27.4	1.5–26.2	3.1–28.1
Ulminite	4.6–34.3	8.9–84.8	41.6	4.7–34.3	8.9–84.8	41.6	12.4–86.9	1.7–62.8	2.4–68.4	13.0–86.9	1.7–62.8	2.5–68.4	16.6–48.6	9.5	2.7–98.7	16.6–48.6	19.1	3.0–98.7	9.5–55.1	19.1–55.2
*Humotelinite*	19.5–70.1	37.2–84.8	59.1	20.0–70.1	42.9–84.8	59.1	33.3–90.7	31.3–76.3	20.5–83.3	48.9–90.7	41.0–76.3	20.5–83.3	30.7–51.8	11.0	2.9–98.7	30.7–51.8	22.2	3.2–98.7	11.0–61.1	22.2–62.1
Attrinite	0.0–5.8	0.0–13.7	0.0	0.0–5.9	0.0–14.2	0.0	0.0–16.1	0.1–3.1	0.0–1.1	0.0–24.1	0.1–4.9	0.0–1.5	0.0–22.9	0.0	0.0–22.8	0.0–22.9	0.0	0.0–22.8	0.0–11.5	0.0–11.5
Densinite	0.0	0.0	0.0	0.0	0.0	0.0	0.0	0.0	0.0	0.0	0.0	0.0	0.0	0.0	0.0	0.0	0.0	0.0	0.0	0.0
*Humodetrinite*	0.0–5.8	0.0–13.7	0.0	0.0–5.9	0.0–14.2	0.0	0.0–16.1	0.1–3.1	0.0–1.1	0.0–24.1	0.1–4.9	0.0–1.5	0.0–22.9	0.0	0.0–22.8	0.0–22.9	0.0	0.0–27.4	0.0–11.5	3.1–28.1
Phlobaphinite	9.4–24.4	0.0–17.3	4.7	9.4–25.0	0.0–18.2	4.7	0.8–9.6	8.7–13.2	0.7–12.3	0.8–10.1	9.0–13.7	0.7–12.4	4.5–58.4	25.8	0.0–53.6	4.5–58.4	52.2	0.0–58.9	4.7–31.5	4.7–52.2
Porigelinite	4.2–26.0	1.3–9.6	12.4	4.2–26.7	1.3–10.2	12.4	0.9–4.8	2.9–7.6	0.7–42.8	0.9–4.8	4.6–7.6	0.9–42.8	4.9–10.6	5.1	0.0–19.6	4.9–10.6	10.4	0.0–21.5	3.0–15.6	3.6–15.7
*Humocollinite*	18.3–50.4	5.0–26.1	17.1	18.3–51.7	5.0–28.0	17.1	1.7–12.7	11.7–20.1	2.6–47.0	1.7–15.4	16.6–20.4	2.6–47.0	9.4–69.0	30.9	0.0–73.2	9.4–69.0	62.6	0.0–80.3	8.2–39.2	10.0–62.6
*Huminite group*	**72.5–88.7**	**51.9–93.0**	**76.1**	**72.5–88.7**	**55.4–93.0**	**76.1**	**51.6–92.4**	**46.1–93.1**	**59.6–86.2**	**62.5–92.4**	**61.9–93.1**	**60.2–86.2**	**84.1–99.7**	**41.9**	**71.3–99.2**	**84.1–99.7**	**84.8**	**71.3–99.2**	**41.9–91.9**	**70.3–91.9**
Fusinite	0.0–1.5	0.0–7.8	0.0	0.1–15	0.0–9.0	0.0	0.0	0.0	0.0–0.7	0.0–0.1	0.0	0.0–0.7	0.0	0.0	0.0–0.3	0.0	0.0	0.0–0.3	0.0–1.3	0.0–1.4
Semifusinite	0.1–8.1	0.0–7.5	1.2	0.0–8.3	0.0–7.5	1.2	0.0–2.7	0.0–1.1	0.0–5.3	0.0–2.7	0.0–1.3	0.0–5.3	0.0–1.8	0.7	0.0–2.2	0.0–1.8	1.5	0.0–2.2	0.6–1.6	0.8–2.3
Micrinite	0.0–0.1	0.0	0.0	0.0–0.1	0.0–0.6	0.0	0.0–0.3	0.0–0.2	0.0–0.4	0.0–0.3	0.0–0.3	0.0–0.4	0.0	0.0	0.0–0.0	0.0	0.0	0.0–0.0	0.0–0.1	0.0–0.1
Macrinite	0.0	0.0	0.0	0.0	0.0	0.0	0.0–0.3	0.0	0.0–0.1	0.0–0.3	0.0–0.1	0.0–0.1	0.0	0.1	0.0–4.3	0.0–0.1	0.2	0.0–4.3	0.0–1.6	0.0–1.1
Secretinite	0.0–0.5	0.0	0.0	0.0–0.5	0.0	0.0	0.0	0.0–0.2	0.0	0.0	0.0–0.3	0.0	0.0	0.0	0.0–0.0	0.0	0.0	0.0–0.0	0.0–0.1	0.0–0.1
Funginite	0.0–3.1	0.0–4.9	0.7	0.0–3.1	0.0–5.0	0.7	0.1–0.9	0.1–1.9	0.5–1.8	0.1–0.9	0.1–1.9	0.5–1.9	0.0–1.6	4.7	0.0–2.1	0.0–1.6	9.6	0.0–2.2	0.1–4.7	0.3–3.3
Inertodetrinite	0.0–0.3	0.0–1.0	0.2	0.0–0.3	0.0–1.0	0.2	0.0	0.1–0.3	0.0–0.5	0.0–0.2	0.0–0.3	0.0–0.5	0.0	0.0	0.0–0.4	0.0–0.1	0.0	0.0–0.4	0.0–0.3	0.0–0.3
*Inertinite group*	**0.6–10.4**	**0.0–10.5**	**2.0**	**0.6–10.7**	**0.0–11.1**	**2.0**	**0.1–1.6**	**0.1–3.3**	**1.2–9.2**	**0.1–1.7**	**0.1–3.4**	**1.6–9.2**	**0.0–3.7**	**5.5**	**0.0–8.2**	**0.0–3.7**	**11.2**	**0.0–8.2**	**1.4–5.5**	**1.4–5.9**
Sporinite	0.0–0.8	0.0–0.2	0.4	0.0–0.8	0.0–0.2	0.4	0.3–6.1	0.0–3.7	0.0–6.3	0.1–6.4	0.0–3.8	0.0–6.3	0.1–1.6	0.1	0.0–3.0	0.1–1.6	0.2	0.0–3.0	0.0–2.2	0.0–2.3
Cutinite	0.1–3.9	0.0–13.4	7.7	0.1–3.9	0.0–13.4	7.7	0.4–7.4	0.0–11.4	0.0–21.3	0.4–7.8	0.0–11.5	0.0–12.8	0.0–0.7	0.0	0.0–5.7	0.0–0.7	0.0	0.0–5.7	0.0–7.7	0.0–7.7
Resinite	0.1–11.0	1.3–29.0	3.7	0.1–11.0	1.5–31.0	3.7	0.6–5.5	0.5–4.4	3.6–19.1	0.6–5.5	0.5–4.5	2.4–20.6	0.0–4.3	0.7	0.3–6.7	0.0–4.3	1.5	0.3–6.7	0.7–11.0	1.5–11.4
Alginite	0.0	0.0	0.0	0.0	0.0	0.0	0.0	0.0	0.0	0.0	0.0	0.0	0.0	0.0	0.0	0.0	0.0	0.0	0.0	0.0
Liptodetrinite	0.0–3.1	0.3–3.6	6.1	0.0–3.1	0.3–3.8	6.1	0.7–12.4	0.8–6.6	1.2–13.2	1.4–13.0	1.3–6.7	1.3–13.2	0.1–4.5	0.6	0.1–2.9	0.1–4.5	1.2	0.1–2.9	0.6–6.1	1.0–6.1
Suberinite	0.1–6.0	0.0–5.0	1.9	0.1–6.0	0.0–5.8	1.9	0.4–8.0	2.0–5.4	0.3–6.6	0.4–8.0	2.0–8.5	0.0–6.6	0.0–0.2	0.2	0.0–3.5	0.0–0.2	0.4	0.0–3.5	0.1–3.9	0.1–4.9
Exsudatinite	0.0–10.9	0.0–8.9	2.0	0.0–10.9	0.0–9.5	2.0	0.1–4.1	0.6–6.2	0.2–6.1	0.2–4.1	0.6–8.0	0.2–6.2	0.1–0.9	0.3	0.0–6.4	0.1–0.9	0.6	0.0–6.4	0.3–4.0	0.5–4.9
*Liptinite group*	**0.3–18.4**	**7.0–41.5**	**21.8**	**0.3–18.4**	**7.0–44.4**	**21.8**	**7.5–34.2**	**6.8–34.2**	**10.2–36.6**	**7.5–35.98**	**6.8–34.7**	**10.2–37.0**	**0.3–12.2**	**2.0**	**0.8–20.5**	**0.3–12.2**	**4.0**	**0.8–20.5**	**2.0–25.3**	**4.0–26.3**
Mineral matter	0.0–2.5	0.0–13.3	0.0	–	–	–	0.0–33.6	0.0–36.1	0.0–26.4	–	–	–	0.0	50.6	0.0–8.9	–	–	–	0.0–50.6	–
IV FACTOR	–	–	–	0.7–12.1	0.0–13.4	2.6	–	–	–	0.1–5.5	0.1–5.2	1.9–12.3	–	–	–	0.0–3.54	11.7	0.0–10.3	–	2.0–11.7

Coal section	MC 01						MC 12						MC 05						Avg	Avg
Seams	01/04	01/02–01/03	01/01	01/04	01/02–01/03	01/01	T	M	B	T	M	B	05/03	05/02	05/01	05/03	05/02	05/01		
Microlithotypes	(vol%)			(vol%, cmf)			(vol%)			(vol%, cmf)			(vol%)			(vol%, cmf)			(vol%)	(vol%, cmf)
Humite	4.5–28.9	5.6–36.6	5.5	5.1–28.9	6.5–36.6	5.5	5.2–68.1	5.7–40.2	1.8–12.5	4.6–68.2	6.0–40.2	1.8–12.5	25.7–96.5	22.9	5.6–96.3	25.7–96.5	71.7	29.0–96.3	5.5–61.1	1.8–12.5
Liptite	0.0–0.6	0.0–3.9	0.0	0.0–0.6	0.0–4.4	0.0	0.0–1.5	0.0	0.0–3.8	0.0–1.7	0.0	0.0–4.3	0.0–0.3	0.6	0.0–3.9	0.0–0.3	1.9	0.0–0.5	0.0–0.8	0.0–4.3
Inertite	0.0–1.3	0.0–2.4	0.0	0.0–1.3	0.0–3.5	0.0	0.0–0.3	0.0–0.5	0.0–0.7	0.0–0.5	0.0–1.1	0.7–0.7	0.0–0.4	1.2	0.0–5.1	0.0–0.4	3.9	0.0–5.1	0.0–1.3	0.7–0.7
Clarite	55.6–88.1	39.5–86.3	88.7	55.6–88.1	57.1–89.7	88.7	19.4–80.8	37.7–80.8	50.3–88.2	31.7–86.2	59.7–84.7	74.5–88.7	3.3–63.5	3.5	3.4–76.4	3.3–63.5	10.9	3.4–63.0	3.5–88.7	74.5–88.7
Durite	0.0–7.5	0.0–6.7	0.0	0.0–8.4	0.0–6.7	0.0	0.0–0.2	0.0–0.2	0.1–1.4	0.0–0.2	0.0–0.4	0.1–1.4	0.0–0.7	3.1	0.0–3.6	0.0–0.7	9.6	0.0–0.8	0.0–3.1	0.1–1.4
Huminertite	0.0–1.6	0.0–1.1	0.1	0.0–1.6	0.0–1.3	0.1	0.0	0.0	0.0–0.1	0.0	0.0	0.0–0.1	0.2–1.3	0.7	0.0–3.0	0.2–1.3	1.9	0.0–3.0	0.0–0.9	0.0–0.1
Duroclarite	1.4–13.7	0.0–14.5	5.7	1.4–13.7	0.0–17.0	5.7	0.1–8.4	0.1–8.4	1.1–18.7	0.1–7.2	0.1–8.8	1.7–18.7	0.0–7.8	0.0	0.0–8.2	0.0–7.8	0.0	0.0–8.2	0.0–8.5	1.7–18.7
Clarodurite	0.0–0.5	0.0–0.3	0.0	0.0–0.6	0.0–0.3	0.0	0.0	0.0	0.0–0.2	0.0	0.0	0.1–0.2	0.0	0.0	0.0–0.1	0.0	0.0	0.0–0.1	0.0–0.1	0.1–0.2
Huminertoliptite	0.0–1.7	0.0–1.5	0.0	0.0–1.9	0.0–1.5	0.0	0.0	0.0	0.0–2.8	0.0–2.0	0.0	0.0–2.8	0.0–0.4	0.0	0.0–0.8	0.0–0.4	0.0	0.0–1.3	0.0–0.7	0.0–2.8
Carbominerite	0.0–11.0	0.0–37.5	0.0	–	–	–	0.1–74.6	0.0–4.8	0.0–43.2	–	–	–	0.0	67.9	0.0–14.5	–	–	–	0.0–67.9	–

Significant values are in bold.

IV factor is also shown.

*T* top, *M* middle, *B* bottom, *IV factor* inertinite–huminite/vitrinite factor by (inertinite × 100)/(inertinite + huminite).

The huminite/vitrinite of the studied coals ranged from 0.39 to 0.49 Ro%. The accuracy of the thermal maturity assessment is also supported by the percentage of non-hydrocarbon compound (65%) and biomarker distributions^24, 25^.

### Palynological composition

**Figure 4:**
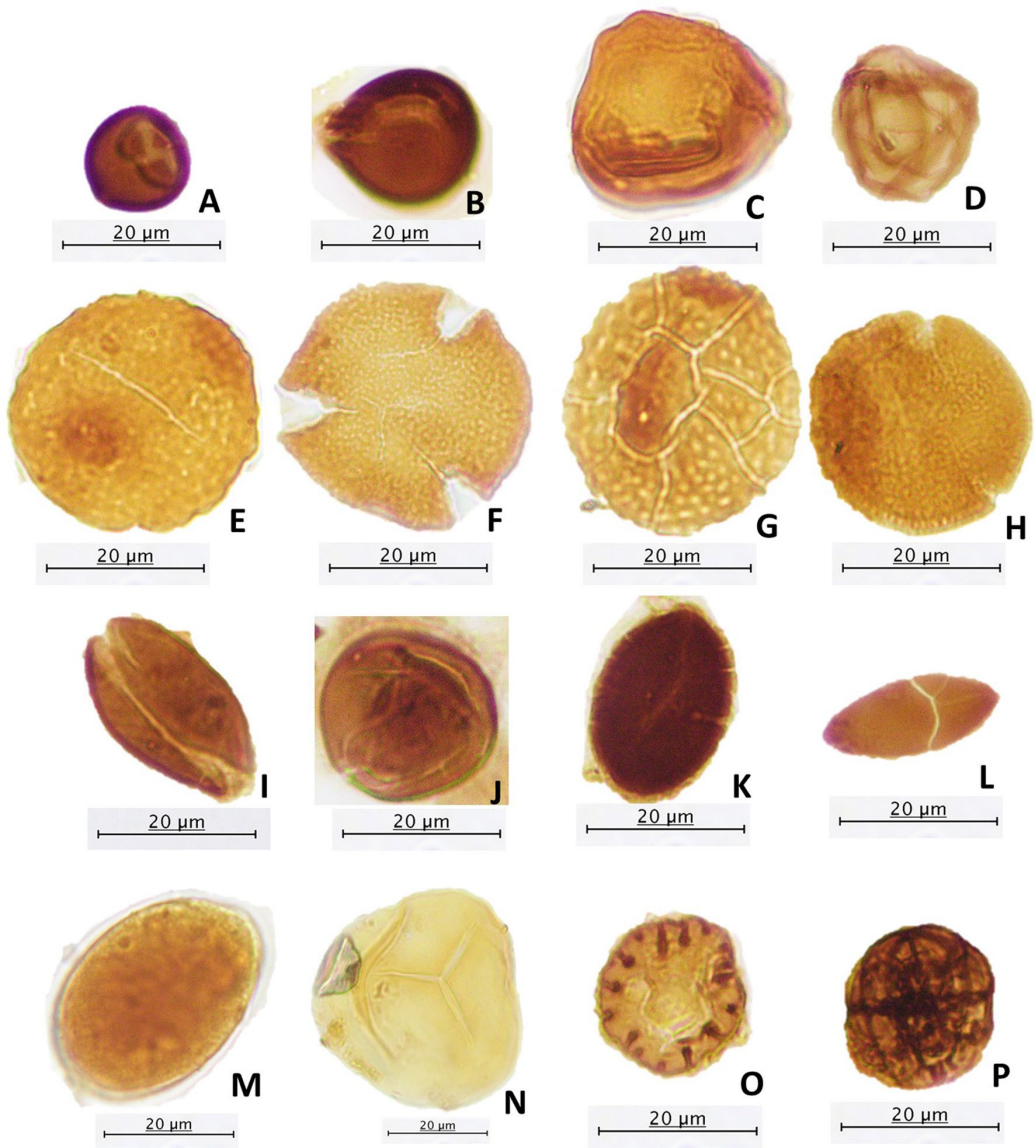
Some of the pollen and spores recovered from the Mukah coal samples being studied. (**A**, **B**, **D**, **I**–**M**, **O**, **P**) Fungus, (**C**) *Casuarina* type, (**E**) *Chepalomappa*, (**F**) *Brownlowia* type, (**G**) ?*Chepalomappa* type, (**H**) *Blumeodendron*, (**N**) *Acrostichum aureum*.

**Figure 5:**
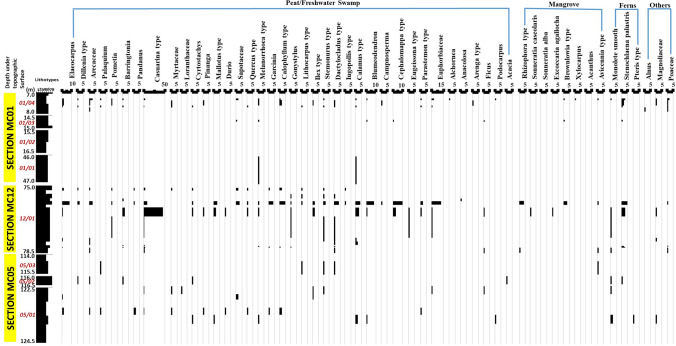
Vertical distribution of different palynotaxas recovered in the Mukah coal samples being studied. The palynomorphs have been grouped into peat/freshwater swamp, mangrove, ferns, and others. The coal lithotypes are also shown.

Twenty-four of the 45 analyzed coal samples contained abundant and well-preserved palynomorphs, with some depicted in Fig. 4, and their vertical distribution shown in Fig. 5. The distribution of the preserved palynomorphs were relatively sparse with several moderately rich coal seams. The total assemblage was dominated by angiosperm pollen, particularly from the families Casuarinaceae (Fig. 4c), Euphorbiaceae (Fig. 4e, g), Acanthaceae, Fabaceae, Tiliaceae (Fig. 4f), Elaeocarpaceae, Arecaceae, Fagaceae, Sapotaceae, Guttiferae, Sonneratiaceae, Crypteroniaceae, Verbenaceae, Anacardiaceae, Palmae, and Myrtaceae. The conifer pollen (gymnosperm) in the assemblage was represented by small amounts of the *Podocarpus* species. The spores of *Stenochlaena palustris*, *Acrostichum aureum* (Fig. 4n), and monolete smooth were also found in relatively small amounts in most of the samples, which are ascribed to the families Dennstaedtiaceae and Filicales. The herbs of Magnoliaceae and Poaceae as well as Alnus from montane were recovered from the studied samples and are of great importance. Pollen of the *Rhizophora* type, *Sonneratia caseolaris*, *Sonneratia alba*, *Excoecaria agallocha*, *Brownlowia* type (Fig. 4f), *Xylocarpus*, *Acanthus*, and *Avicennia* type were not recovered in any of the samples within section MC05 (Fig. 5). However, a few of these pollens were present in minor quantities in a few samples within sections MC12 and MC01 (Fig. 5). Section MC12 displayed a high frequency of palynomorph assemblages with a dramatic increase in their occurrence, as seen in sample No. 13 and 20 (Fig. 5). Pollen of the *Casuarina* type (Fig. 4c) was found in abundance within the middle section of the seam (No. 20) (Fig. 5). The upper part of section MC01 showed palynomorph assemblages in considerable amounts, with *Stenochlaena palustris*, *Elaeocarpus*, and Arecaceae being the most frequent within this interval (Fig. 5).

### Bulk geochemical data

**Figure 6:**
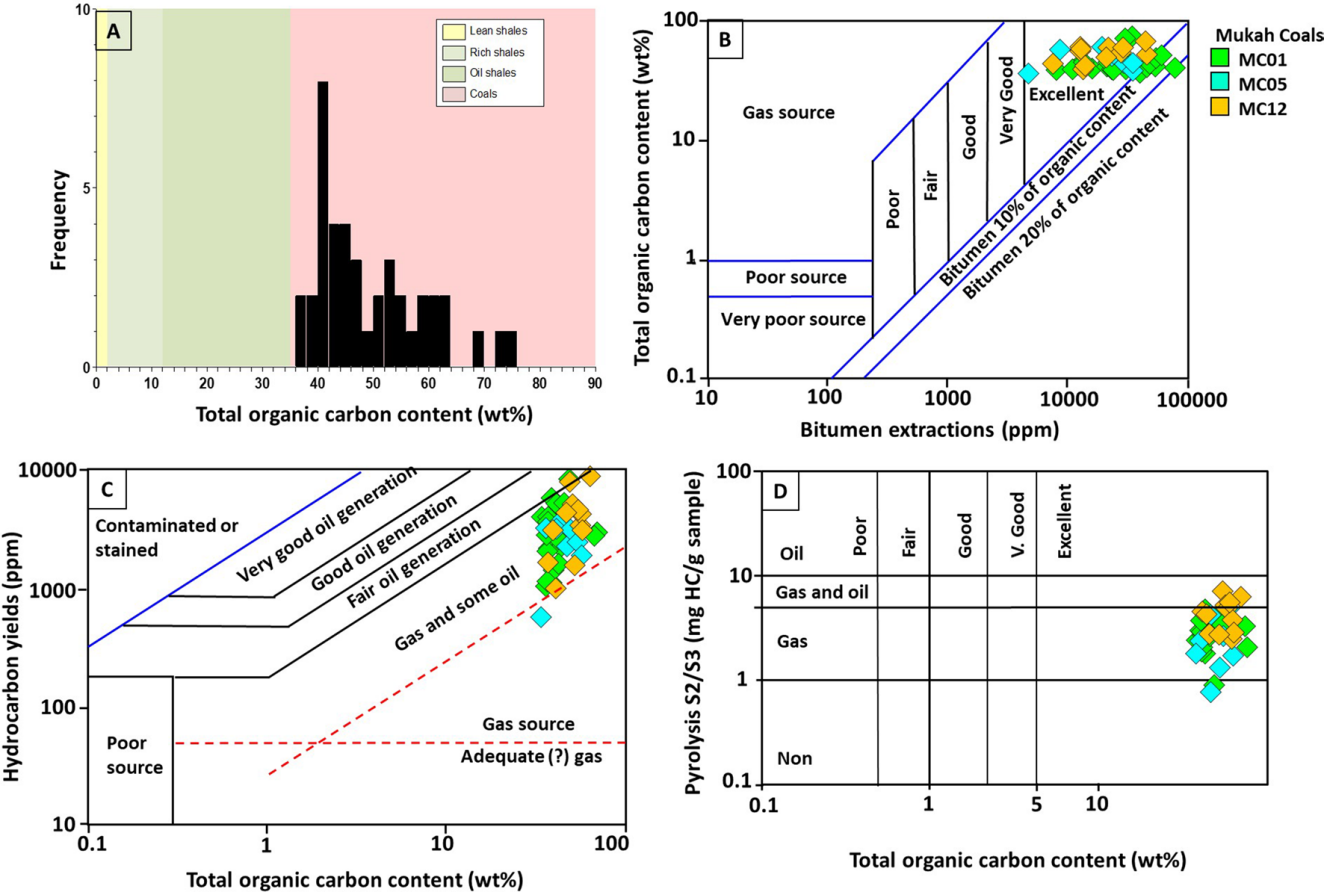
(**A**) Histogram of total organic matter content (TOC), (**B**) pyrolysis *S*_2_/*S*_3_, (**C**) distribution of extractable organic matter (EOM) and (**D**) hydrocarbon yield (HC), showing source potential rating and hydrocarbon source rock richness for the selected samples from Mukah coals, Sarawak.

The results of the bulk organic geochemical studies of the Mukah coals are reported in Table 2. High organic carbon values with TOC ranging from 37.62 to 75.8 wt% (Fig. 6a) and high quantities of extractable organic matter (EOM) and hydrocarbon yields (HC), exceeding 29,000 and 18,000 ppm, respectively, were recorded (Table 2). There is a good relationship between the high EOM and TOC content, implying that the analyzed coal samples are rich in organic matter (Fig. 6b), hence, are capable of producing substantial gas with little to fair oil product^26^ (Fig. 6c). Table 2 also shows the pyrolysis parameters of the studied coals that were utilized to evaluate the amount and quality of organic matter, along with their potential for HCs. The *S*_1_, *S*_2_, and *S*_3_ yields of the analysed samples were 0.6–3.5, 20.9–108.7, and 9.3–37.6 mg HC/g rock, respectively (Table 2). The high hydrocarbon yield (*S*_2_) content produced of 6 mg HC/g rock (*S*_2_) is the minimum threshold required to be regarded as an efficient source of hydrocarbons^27^. The cross plot between the TOC content and pyrolysis *S*_2_/*S*_3_ yield values showed that the Mukah coals are mainly good to very good source rocks and are both gas- and oil- prone, with high gas generation potential^26^ (Fig. 6d). The *S*_2_ and *S*_3_ yields were also compatible with the TOC content and were used to calculate HI and OI values according to^26^. The hydrogen index (HI) ranged from 124 to 377 mg HC/g rock while the oxygen index (OI) varied from 43 to 161 mg CO_2_/g rock (Table 2).

**Table 2:** Vitrinite reflectance, total organic carbon content (TOC), extractable organic matter (EOM), pyrolysis data, relative percentages of saturates, aromatics and nitrogen–sulphur–oxygen (NSO) compounds of EOM, ultimate analysis data and atomic ratios (dried-ash free).

Sample ID	VR (R_o_%)	TOC (wt%)	Pyrolysis data (Rock–Eval)							Bitumen extraction and LCC (ppm of whole rocks)						Ultimate analysis (wt%)					Atomic ratios (daf)				S/TOC
			*S1*	*S2*	*S3*	HI	OI	PI	T_max_	EOM	Sat	Aro	NSO	HC	Sat/Aro	C	H	N	S	O	H/C	O/C	C/N	S/C	
**MC01**																									
01/04	0.43–0.44	37.9–42.8	1.1–2.2	46.6–78.5	25.8–30.9	286–377	146–158	0.02–0.03	413–416	9366–29,788	388–14,328	1019–3227	7959–14,938	1407–18,429	0.12–3.49	49.12–67.41	2.89–4.30	1.62–2.45	0.34–2.35	25.40–51.86	0.84–1.03	0.29–0.69	27.73–37.63	0.00–0.02	0.01–0.05
01/02–01/03	0.39–0.45	39.9–48.2	0.6–1.7	33.4–68.8	11.3–37.5	139–339	76–156	0.02	413–420	3953–12,692	419–590	855–2066	2635–10,036	1317–2657	0.29–0.56	26.45–66.92	1.26–3.58	1.29–2.39	0.42–1.30	26.17–69.53	0.77–1.05	0.61–1.97	23.86–30.34	0.00–0.01	0.01–0.03
01/01	0.40–0.45	40.8–46.6	1.3–1.8	43.9–82.6	13.4–31.2	240–363	73–140	0.01–0.03	408–426	6291–19,367	632–1377	1332–4367	44,327–13,623	1964–5743	0.32–0.46	36.53–64.46	3.05–4.22	1.59–2.64	0.34–1.33	27.35–57.94	0.69–1.10	0.32–1.19	23.17–30.78	0.00–0.01	0.01–0.03
**MC12**																									
T	0.42–0.44	37.6–62.3	0.6–1.6	20.9–84.4	11.7–28.1	128–286	60–90	0.01–0.02	418–421	2467–8634	254–935	508–1949	1325–6352	763–2884	0.47–0.64	22.90–63.70	1.58–3.70	1.25–2.88	0.35–0.67	30.13–73.59	0.65–0.83	0.35–2.41	21.39–31.44	0.00	0.01–0.02
M	0.43–0.46	45.2–61.4	1.0–2.8	45.2–92.7	16.4–30.0	200–332	63–98	0.01–0.03	417–426	3730–11,836	443–2329	835–3350	2452–6157	1277–5679	0.34–0.7	30.10–68.47	2.00–3.86	1.55–2.79	0.18–0.29	24.59–66.17	0.68–0.86	0.27–1.65	22.72–28.68	0.00	0.00
B	0.43–0.49	43.4–62.7	1.2–2.9	39.8–108.7	9.3–22.4	183–364	43–80	0.02–0.06	409–422	6015–12,312	695–1942	1216–7231	859–15,643	1911–5156	0.44–1.19	52.28–73.23	4.07–4.39	2.01–3.09	0.24–0.31	19.60–41.08	0.67–1.01	0.20–0.59	30.03–34.20	0.00	0.00–0.01
**MC05**																									
05/3	0.45–0.47	40.2–75.8	0.9–2.3	49.9–64.7	13.5–31.5	248–326	67–159	0.01–0.04	421–422	10,369–14,317	825–1407	2621–1889	7073–10,871	3296–3446	0.31–0.74	31.13–69.66	3.08–3.92	1.72–2.77	0.20–0.57	23.08–56.87	0.67–0.97	0.25–1.12	25.91–29.31	0.00–0.01	0.01
05/02	0.43–0.46	39.7–53.2	0.8–3.5	28.6–87.5	13.4–37.2	124–334	58–161	0.01–0.05	416–423	12,466–23,647	853–3034	2675–5876	8454–	3711–8910	0.31–0.51	59.80–64.91	4.19–4.78	2.01–2.83	0.20–0.57	29.46–33.57	0.79–0.97	0.32–0.42	26.13–34.67	0.00	0.00–0.01
													14,737												
05/01	0.41–0.47	52.1–54.5	0.9–2.1	49.7–80.3	30.3–37.6	191–295	111–144	0.02–0.03	416–417	10,199–10,560	736–988	1963–2653	6919–7500	2699–3642	0.35–0.37	65.05–69.97	4.01–4.15	2.77–2.90	0.25–0.29	22.84–27.78	0.69–0.77	0.24–0.32	27.38–28.20	0.00	0.00–0.01
Min. value	0.39	37.6	0.6	20.9	9.3	124	43	0.01	408	2467	254	508	859	763	0.12	22.9	1.26	1.25	0.18	19.6	0.65	0.2	21.39	0.00	0.00
Max. value	0.49	75.8	3.5	108.7	37.6	377	161	0.06	426	29,788	14,328	7231	15,643	18,429	3.49	73.23	4.78	3.09	2.35	73.59	1.1	2.41	37.63	0.02	0.05
Average	0.44	54.6	1.7	68.9	25.9	297	114	0.03	463	11,634	1410	2711	7513	4121	0.52	74.44	4.88	2.98	0.62	49.22	1.12	0.83	38.16	0.00	0.01

*VR* vitrinite reflectance (R_o_%), *TOC* total organic carbon content, wt%, *S*1 volatile hydrocarbon (HC) content, mg HC/g rock, *S*2 remaining HC generative potential, mg HC/g rock, *S3* carbon dioxide yield, mg CO_2_/g rock, *T*_*max*_ temperature at maximum of *S*2 peak, *HI* Hydrogen Index = *S*2 × 100/TOC, mg HC/g TOC, *OI* Oxygen Index = *S*3 × 100/TOC, mg CO_2_/g TOC, *PI* Production Index = *S*1/(*S*1 + *S*2), *EOM* extractable organic matter, *Sat* saturates, *Aro* aromatic, *NSO* nitrogen–sulphur–oxygen, *HC* hydrocarbon.

T_max_, the temperature in the *S*_2_ peak, at which maximum hydrocarbon yield and production index (PI) values of coal samples were obtained via Rock–Eval pyrolysis, ranged between 408 and 426 °C and 0.01 to 0.06, respectively (Table 2).

### Elemental analysis

Carbon, oxygen, hydrogen, nitrogen, and sulfur concentrations of the studied coal samples were found to be within the range of 22.90–73.23 wt%, 19.60–73.59 wt%, 1.26–4.78 wt%, 1.25–3.09 wt%, and 0.18–2.35 wt%, respectively (Table 2). The H/C, O/C, C/N, and S/C ratios based on a dried-ash-free basis were calculated and are represented by the mean value of 1.12 wt%, 0.83 wt%, 38.16 wt%, and 0.00 wt%, respectively.

### Molecular composition

**Figure 7:**
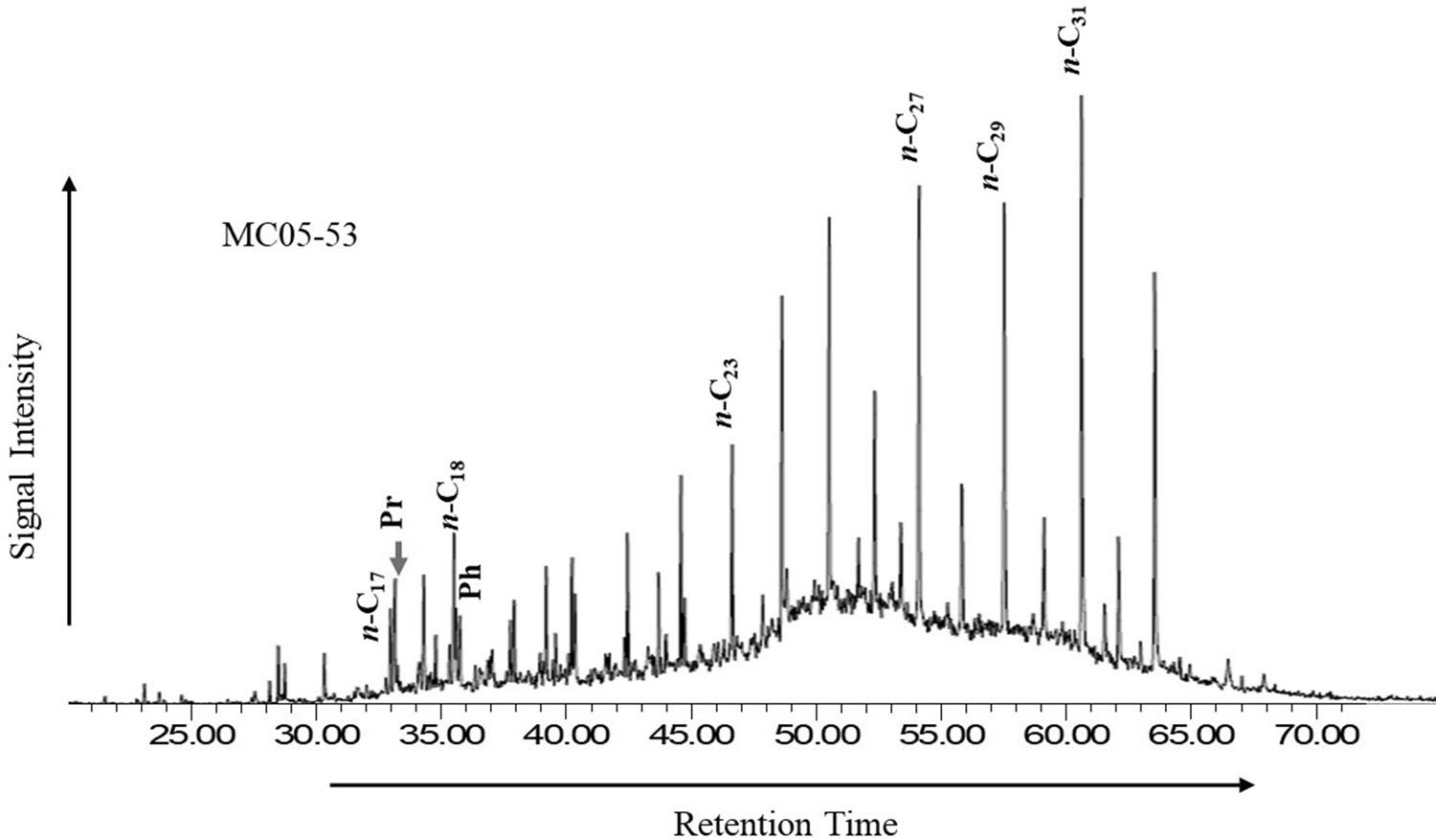
Example of *n*-alkanes (*m/z* 85) distribution of the studied Mukah coals. Numbers represent the carbons in *n*-alkanes chain.

Hydrocarbon distributions of *n*-alkane molecules together with pristane (Pr) and phytane (Ph) in the analyzed coal samples displayed a bimodal distribution along the entire C_16_-C_33_ range (Fig. 7). The *n*-C_27_, *n*-C_29_, and *n*-C_31_ compounds show the highest abundance, suggesting a contribution of higher molecular plant compounds, resulting in a relatively high CPI range between 0.86 and 3.66 (mean 1.57). In addition, Pr and Ph isoprenoid hydrocarbons predominate in most coal sample chromatograms aside from *n*-alkanes (Fig. 7), with Pr being more abundant than Ph, and the ratio of Pr/Ph ranging from 1.00 to 3.67 (mean 2.10). Data also showed that the Pr/Ph ratio of most samples (80%) was higher than 1.0, whereas the remaining 20% was higher than 3.0. The ratio of Pr/*n*-C_17_ varied from 0.31 to 6.67, with an average of 1.93, and Ph/n-C_18_ ratio ranging from 0.14 to 1.00, with an average of 0.40.

**Figure 8:**
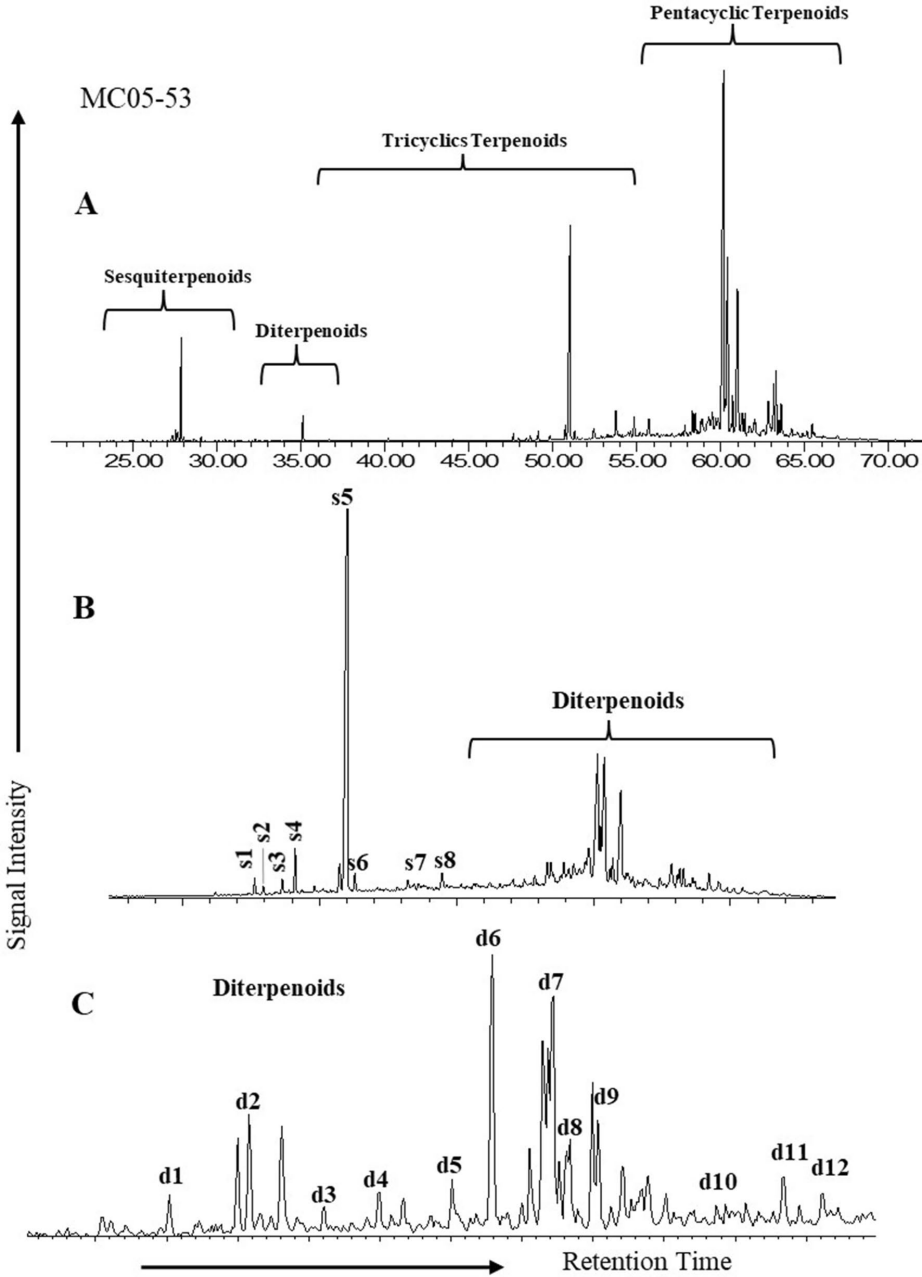
Examples of (**A**) total ion chromatogram (TIC), (**B**) sesquiterpanes (*m/z* 123), and (**C**) diterpanes (*m/z* 123) distributions of the analysed samples from the Mukah coals.

Bicyclic sesquiterpanes (C_14_ to C_16_), 4β (H)-Eudesmane, 8β (H)-Drimane and 8β (H)-Homodrimane compounds have been identified in the *m/z* 123 mass chromatograms (Fig. 8b) in most of the analysed coal samples. These coals were mainly dominated by 4β (H)-Eudesmane, while bicyclic sesquiterpanes, drimanes, and homodrimane were found in low concentrations.

Diterpanes (*m/z* 123) distributions were performed on the selected samples. Tricyclic diterpane iso-Pimarane has been found with tetracyclic diterpanes such as 16β-Phyllocladane, *ent*-Beyerane, 16α-Kaurane, 16α-Phyllocladane and 16β-Kaurane (Fig. 8c), along with other diterpanes including C_18_ and C_20_ diterpane, demethylated ent-beyerane, and sandaracopimarane. Abietane was not detected in the GCMS as the compound and co-elution with other diterpene compounds made it difficult to be identified. Diterpanes ratio (R_dit_) ranged from 1.13 to 1.59 (mean 1.32). Meanwhile, samples from the lower section of the Mukah coals (MC05-08, 53) had a R_dit_ value of 1.59 (> 1.5 R_dit_).

**Figure 9:**
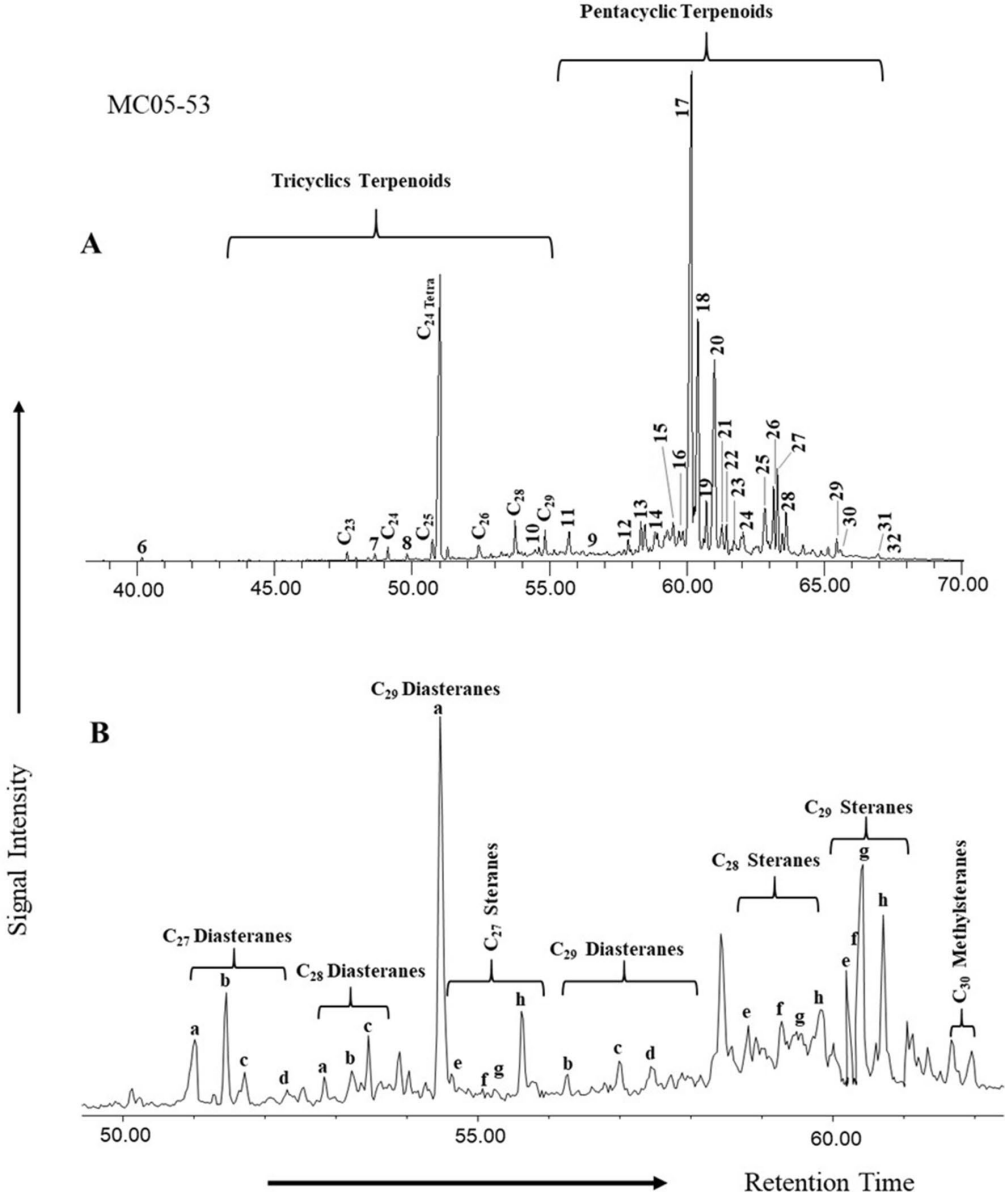
Representative (**A**) tricyclic and pentacyclic terpanes (*m/z* 191) and (**B**) regular steranes and diasteranes distributions (*m/z* 217) in the Mukah coal samples.

The tetracyclic terpanes found include degradation products of oleanene, ursene, and lupane (Fig. 9a). In general, hopanes were found in significant amounts over tricyclic terpanes (*m/z* 191; Fig. 9a), predominantly over C_29_-norhorhopane, Olean-12-ene + Ursa-2,12-diene, C_30_-hopane, and C_31_-C_32_ homohopanes in most of the samples. The greater frequency of C_29_-norhorhopanes in the studied samples (Fig. 9a) led to high C_29_/C_30_ hopane values of greater than 1 (1.17–4.50), as reported in Table 3. The homohopanes of C_31_-C_32_ from the examined samples were dominated by C_31_ and decreased toward C_32_ homohopanes. The calculated ratio of C_31_R/C_30_ hopane was high for the low-rank coals of Mukah. 17α (H)-trisnorneohopane (Tm) and 18α (H)-trisnorneohopane (Ts) occurred at low concentrations, with Tm concentration being slightly higher than Ts, in all analysed Mukah coals. The ratios of Ts/Tm, Tm/Ts, and Ts/(Ts + Tm) were subsequently calculated, resulting in Ts/Tm values being greater than 0.31 (mean 10.67), Tm/Ts values > 0.67 (mean 1.71), and Ts/(Ts + Tm) > 0.24 (mean 0.39).

**Table 3:** Molecular composition of the studied coal. compound numbers are stated in Appendix I.

Sample ID	Pr/Ph	Pr/C_17_	Ph/C_18_	CPI	R_dit_	C_24_T/C_24_Te	C_25_T/C_26_T	C_23_T/C_24_T	C_24_T/C_24_T	C_23_T/C_24_Te	C_23_T/C_24_T	C_24_Te/C_26_T	Ts/Tm	Tm/Ts	Ts/(Ts + Tm)	C_29_/C_30_	C_30_M/C_30_H	HCR_31_/HC_30_	C_32_ S/(S + R)	C_31_ S/(S + R)	C_30_M/C30H	C_27_/C_29_R	C_29_/C_27_R	C_27_	C_28_	C_29_
**MC01**																										
01/04	1.50–2.22	0.52–0.91	0.22–0.29	1.22–1.88	1.40–1.40	0.08–5.00	0.25–5.00	0.40–1.00	0.20–13.00	0.08–2.50	0.01–1.00	1.00–11.00	0.67–1.00	1.00–1.50	0.40–0.50	2.74–4.31	0.13–0.31	0.07–0.24	0.37–0.46	0.50–0.81	0.13–0.31	0.16–0.36	2.75–6.29	11.97–24.38	6.49–16.39	66.98–75.21
01/02–01/03	1.00–3.00	0.51–6.67	0.22–1.00	1.05–2.33	–	0.27–11.15	4.00–30.00	0.01–0.83	0.09–3.67	0.15–0.54	0.01–0.83	5.20–22.00	0.50–2.00	1.09–2.00	0.33–0.48	2.80–4.50	0.07–0.64	0.07–0.36	0.35–0.54	0.55–0.82	0.07–0.64	0.12–0.35	2.82–8.62	9.25–24.38	5.80–16.47	68.83–79.74
01/01	1.44–3.67	0.42–3.67	0.20–0.50	0.86–3.66	1.14–1.14	0.05–1.25	0.58–6.00	0.80–2.00	0.80–22.00	0.09–1.00	0.80–2.00	2.00–16.00	0.33–1.50	0.67–3.00	0.25–0.60	2.12–3.16	0.09–0.26	0.09–0.34	0.28–0.53	0.23–0.76	0.09–0.26	0.19–0.59	1.69–5.18	14.79–34.17	5.23–9.84	57.79–76.63
**MC12**																										
T	1.19	0.43	0.14	1.50	1.13	0.14	1.00	1.00	7.00	0.14	1.00	2.33	0.71	1.40	0.42	2.71	0.10	0.16	0.52	0.47	0.10	0.30	3.33	20.27	12.16	67.57
M	2.79	1.06	0.18	1.23	–	0.03	2.00	2.50	29.00	0.09	2.50	9.67	0.71	1.42	0.41	3.38	0.09	0.38	0.48	0.38	0.09	0.36	2.78	23.05	12.77	64.18
B	3.19–3.67	2.50–2.91	0.31–0.36	1.05–1.89	1.20–1.20	0.11–0.63	0.38–1.67	0.40–2.00	1.60–9.00	0.22–0.25	0.40–2.00	1.50–3.00	0.31–1.11	0.90–3.20	0.24–0.53	3.47–3.67	0.08–0.22	0.14–0.24	0.50–0.56	0.55–0.58	0.08–0.22	0.48–0.49	2.05–2.07	27.25–27.86	15.04–16.25	56.50–57.10
**MC05**																										
05–03	1.83	1.00	0.28	0.97	1.45	0.20	0.54	1.00	5.00	0.20	1.00	2.00	0.62	1.62	0.38	1.71	0.06	0.16	0.51	0.61	0.06	0.25	4.02	18.57	6.84	74.59
05/02	1.00–3.32	0.36–4.20	0.28–0.46	0.89–2.44	–	0.06–0.12	2.00–3.75	0.75–1.75	8.50–17.00	0.09–0.10	0.75–1.75	8.50–11.33	0.50–1.00	1.00–2.00	0.33–0.50	2.75–3.97	0.19–0.48	0.10–0.18	0.52–0.54	0.72–0.73	0.19–0.48	0.21–0.33	3.06–4.69	15.66–19.32	10.86–21.52	59.17–73.48
05/01	1.15–1.60	0.31–1.26	0.23–0.47	1.05–1.49	1.59–1.59	0.03–0.04	1.17–3.00	1.25–2.00	25.50–39.50	0.03–0.08	1.25–2.00	26.33–51.00	0.43–0.80	1.25–2.33	0.30–0.44	2.49–2.89	0.11–0.13	0.07–0.09	0.51–0.53	0.33–0.57	0.11–0.13	0.19–0.22	4.59–5.26	15.03–15.05	5.94–15.86	69.09–79.02
Min. value	1.00	0.31	0.14	0.86	1.13	0.03	0.25	0.01	0.09	0.03	0.01	1.00	0.31	0.67	0.24	1.71	0.06	0.07	0.28	0.23	0.06	0.12	1.69	9.25	5.23	56.50
Max. value	3.67	6.67	1.00	3.66	1.59	11.15	30.00	2.50	39.50	2.50	2.50	51.00	1.50	3.20	0.60	4.50	0.64	0.38	0.56	0.82	0.64	0.59	8.62	34.17	21.52	79.74
Average	2.10	1.93	0.40	1.63	1.32	1.01	4.99	1.13	9.84	0.33	1.11	9.48	0.67	1.71	0.39	3.09	0.20	0.17	0.46	0.62	0.21	0.28	4.17	18.94	10.83	70.23

Pr: pristane; Ph: phytane; CPI: carbon preference index (1): {2(C_23_ + C_25_ + C_27_ + C_29_)/(C_22_ + 2[C_24_ + C_26_ + C_28_] + C_30_)}; R_dit_: (19-norisopimarane + isopimarane + 16α-kaurane)/(ent-beyerane + 16β-phyllocladane + 16α-phyllocladane); C_24_T/C_24_Te: C_24_Tricyclic/C_24_Tetracyclic; Ts: (C_27_ 18α(H)-22,29,30-trisnorneohopane); Tm: (C_27_ 17α(H)-22,29,30-trisnorhopane); C_29_/C_30_: C_29_ norhopane/C_30_ hopane; HCR_31_/HC_30_: C_31_ regular homohopane/C_30_ hopane; C_31_S/(S + R): C_31_Homohopane 22S/(22S + 22R); C_32_S/(S + R) = C_31_Homohopane 22S/(22S + 22R); C_29_/C_27_R = Regular steranes C_29_/C_27_.

The distributions of steranes and diasteranes (*m/z* 217; Fig. 9b) were strongly concentrated. The results indicate a higher concentration of C_29_ sterane (56.50–79.74%), a moderate concentration of C_27_ (7.97–34.17%), and a low concentration of C_28_ (5.23–21.52%) as shown in Table 3.

## Discussions

### Thermal maturity of coal

**Figure 10:**
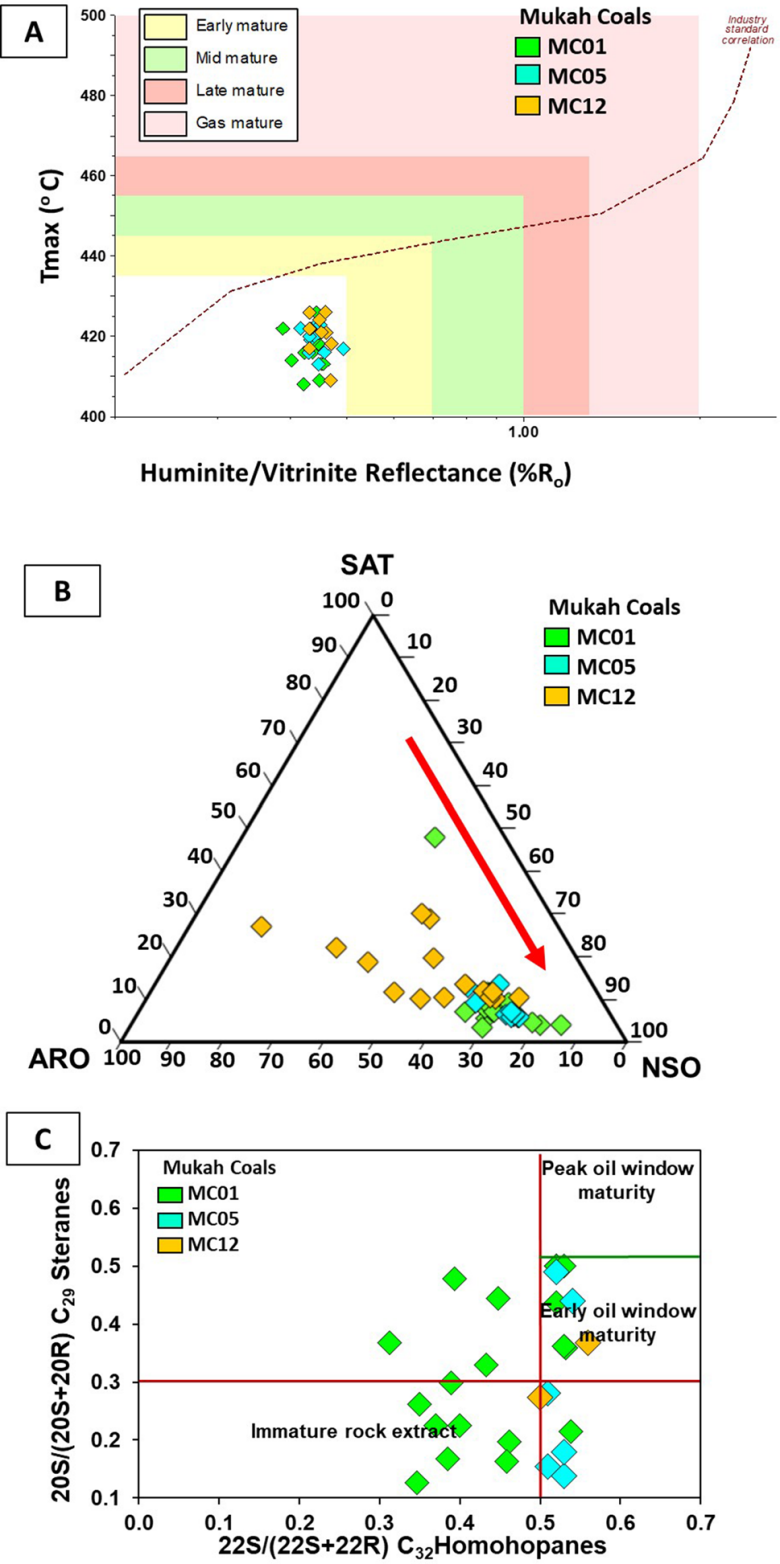
Diagrams illustrate (**A**) T_max_ versus huminite/vitrinite reflectance measurement (%Ro), (**B**) ternary diagram of saturates (SAT), aromatic (ARO) and NSO compound and (**C**) cross plot of 20S/(20S + 20R) C_29_ steranes against 22S/(22S + 22R) C_32_ homohopanes, showing their distribution and the thermal maturity level of the studied Mukah coals samples.

The huminite/vitrinite values indicate a lignite to sub-bituminous B type for the Mukah coals. T_max_ values differ from 409 to 426 °C (Table 2), showing immature organic matter^27^. The results are consistent with the reflectance values of the T_max_ versus huminite/vitrinite reflectance plots (Fig. 10a). The high concentration of NSO compound (65%) obtained from the EOM supports the low to moderate thermal maturity of the Mukah coals (Fig. 10b).

Concerning the biomarker distributions of homohopanes C_31_ to C_32_ (*m/z* 191) and their frequencies, most of the samples display a relative dominance of the “R” over the “S” epimers, indicating a low to moderate degree of thermal maturity^24, 25^. The ratio of C_32_ homohopanes 22S/(22S + 22R) values range from 0.28 to 0.56 (mean 0.46), suggesting an increase in thermal maturity from the upper to lower coal seams of the Mukah coals. The ratios of C_29_ ββ/(ββ + αα) sterane range from 0.03 to 0.50 (mean 0.31) indicating low thermal maturity. The cross plot of 20S/(20S + 20R) steranes C_29_ and C_32_ 22S/(22S + 22R) homohopanes (Fig. 10c) shows that the Mukah coal samples were thermally immature to early mature (oil window), consistent with the n-alkanes distribution, CPI values from odd to even carbon ratios of n-alkanes, and cross plot of Pr/n-C_17_ vs. Ph/n-C_18_^25, 28^. In addition, C_30_-moretane/C_30_-hopane ratios of the analysed coals vary from 0.06 to 0.64 which decreased with increasing thermal maturity^29^, as a result of less stable moretane than hopane. The Ts/Tm ratio was also used as an indicator of maturity, type of organic matter, and lithology^24, 29–32^. In this study, all of the studied samples contained lower Ts content compared to Tm, suggesting low maturity with the Ts/Tm ratio generally increasing as maturity increases from the upper to lower coals seams of the Mukah coals as Ts is thermally more stable than Tm^24, 25, 30^. In the present study, the existence of aliphatic diterpenoids of *ent*-16α(H)-kaurane, 16α(H)-phyllocladane along with sesquiterpanes is similar to the study on low maturity Gondwana coal formations from South Karanpura and Raniganj Sub-basins, India by^33^.

### Paleomires condition

**Figure 11:**
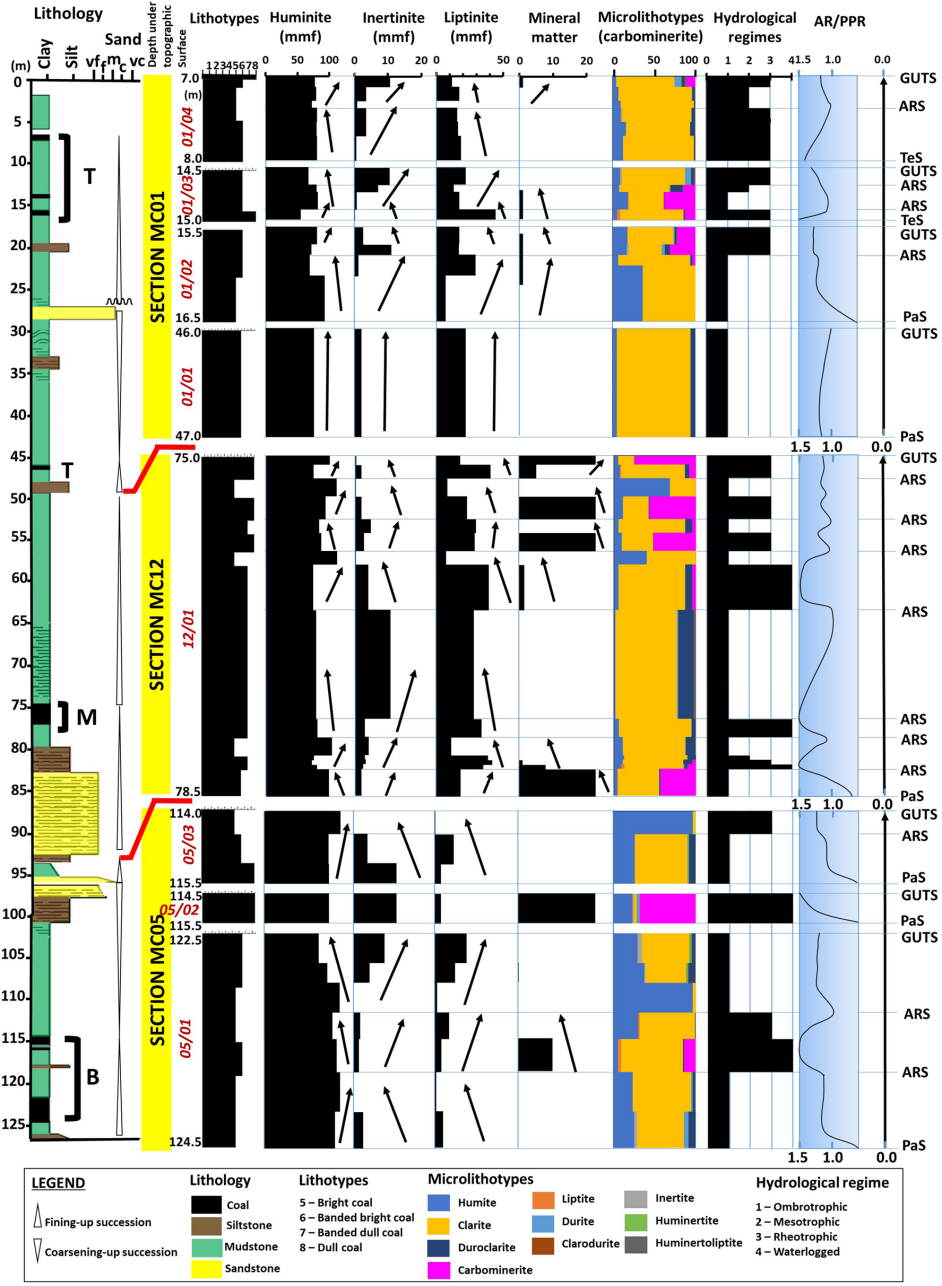
Vertical compositional variations shown are vitrinite (mineral free), inertinite (mineral free), liptinite (mineral free), detrital-mineral matter, and microlithotypes, and interpreted accommodation curves for the Mukah coals, Balingian Formation. On the coal facies diagrams, the solid line represents an accommodation reversal surface (ARS).

The vertical profile shows the Mukah coals enriched with brighter coals, huminite maceral, and clarite from the base to the top seams (Figs. 2, 11). The richness of huminite maceral implies the existence of oxygen-deficient and water-saturated conditions in the precursor mire^34, 35^. The apparent lack of dull coal and extremely low inertinite content (< 10%) further suggests minimal wildfires or burning and oxidation of the peat^36, 37^. The uncommon occurrences of pyrite and low sulfur content in the coals suggest peat accumulated in freshwater mires with little or no marine influence. Furthermore, the abundance of 80% angiosperm pollen supports the depositional setting of the Mukah coals as freshwater peat swamp. Generally, arborescent plant types are predominant, suggesting the characteristic of typified bog facies in the Mukah coals.

**Figure 12:**
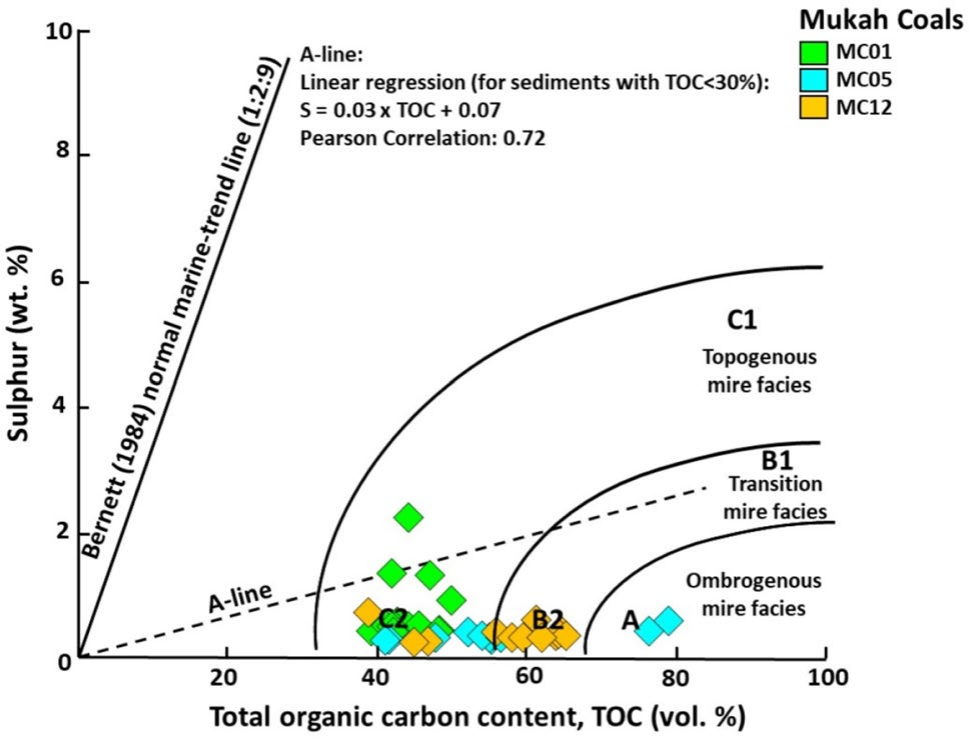
A plot of sulfur (wt%) versus TOC (wt%) for the analyzed Mukah coals (after Jasper et al., 2010) shows the peat development within topogenous to ombrogenous mire facies.

The plot of S versus TOC (Fig. 12) may further describe the development of peat in freshwater mires within topogenous to ombrogenous mire facies, as suggested by^38^. The vertical changes in hydrological regimes (Fig. 11) under which the precursor mires accumulated indicate that the peat-forming mires began with predominantly ombrotrophic mires and eventually ended in rheotrophic mires because of the moderate to rapid rise of the water table during peat accumulation. In this study, the ombrotrophic condition is also evident by the presence of sesquiterpenoid compounds such as rearranged bicyclic sesquiterpanes (C_14_ to C_16_), 8β (H)-Drimane and 8β (H)-Homodrimane^39, 40^. Temporary development of rheotrophic–ombrotrophic mires may have occurred because of the water table fluctuations. Here, the mires were fed by rainfall and groundwater levels that formed during low to moderate water floods. In most cases, the intervals suggest that waterlogged and rheotrophic origins are related to the occurrence of increased mineral matter, which was probably influenced by the clastic influx from the fluvial input into the mires with rising water table levels. This interpretation is also compatible with the diterpanes ratio (R_dit_) of the Mukah coals which ranged from 1.13 to 1.59 and the majority falling into group 3 for lower (MC12) and upper (MC01) sections, indicating fluctuations in the water table may have occurred in the paleomires^28, 29^. Meanwhile, the lower section of the Mukah coals (MC05-08, 53) fall under group 2 with R_dit_ 1.59 (> 1.5 R_dit_), implying the coals in the middle stratal section were deposited in mires influenced by a high-water table^41, 42^.

Furthermore, the biomarker distribution of normal alkane, isoprenoid, sesquiterpane, diterpene, terpane, and sterane of the saturated HC fraction in the extracted coal samples, as shown in mass fragmentograms of *m/z* 85, *m/z* 123, *m/z* 191 and *m/z* 217 ions (Figs. 7, 8, 9) and their ratios, were examined in this study and utilized to assess organic matter inputs in the source rocks, as well as their probable origin and paleoenvironmental conditions (e.g.^25, 29, 43, 44^).

The normal alkanes have a bimodal distribution, with the highest abundance of waxy alkanes (+ *n*-C_23_) (Fig. 7), indicating that the analyzed coal sediments were home to mixed organic matter along with a high volume of terrigenous OM input. The interference of the mixed organic matter input, with a higher contribution from the terrestrial organic matter into these coal sediments, is validated by the CPI values (Table 3). Coal samples show that CPI values of more than 1.5 (1.52–3.66), whereas other samples have CPI values between 1.05 and 1.22 (Table 3), which therefore further supports the interpretation of a mixture organic matter input, with large amounts of terrestrial organic matter input^29^.

The isoprenoid ratios of Pr/Ph, Pr/*n*-C_17_, and Ph/*n*-C_18_ are widely employed to infer organic matter inputs and their depositional conditions (e.g.^45–48^). Pr/Ph ratios of > 3 (3.00–3.67) indicate oxic environmental settings, while Pr/Ph ratios between 1.00 and 2.90 specify less oxidation (suboxic) settings during deposition. The ratio of Pr/Ph of the analyzed coal samples (1.46–2.46) implies that these sediments most likely received large amounts of terrestrial organic matter input under oxic to moderately oxic (suboxic) conditions.

**Figure 13:**
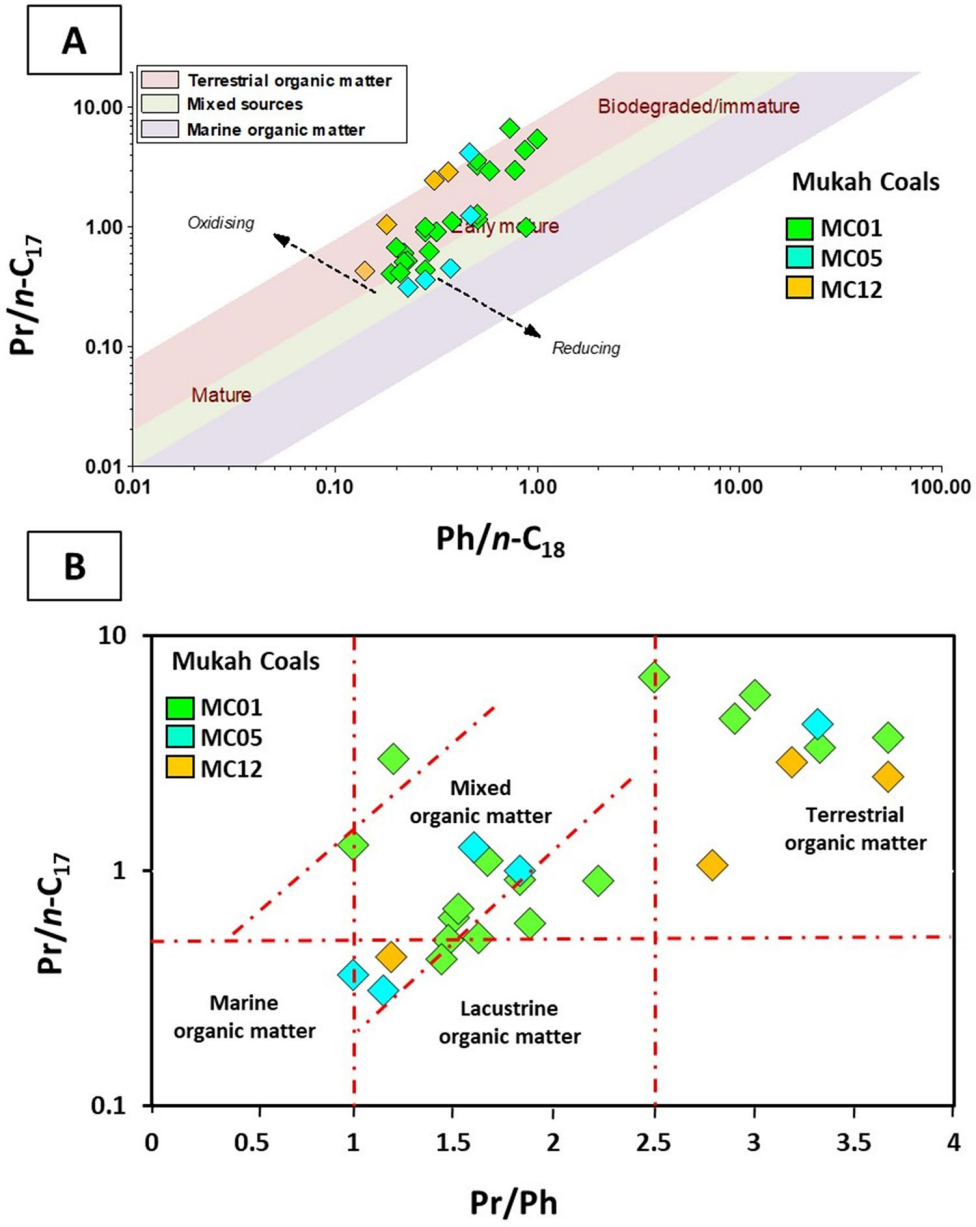
Plots of (**A**) Pristane/*n*-C_17_ versus Phytane/*n*-C_18_ (modified after Lijmbach, 1975) and (**B**) Pristane/*n*-C_17_ versus Pristane/Phytane, showing the source input from a mixed to terrestrial organic matter for the analyzed Mukah coals, Sarawak.

The cross plot of Pr/*n*-C_17_ against Ph/*n*-C_18_^49^ (Fig. 13a) also shows the large contribution of mixed OM with high amounts of terrigenous OM input that was most likely deposited under oxic to moderately oxic (suboxic) environmental conditions. This can be defined from the distribution of the *n*-alkanes and acyclic isoprenoids, which show substantial occurrences of compounds with high molecular weight from *n*-C_23_ upwards in most of the analysed samples, implying that higher plant molecular compounds were the source inputs^27, 29^.

**Figure 14:**
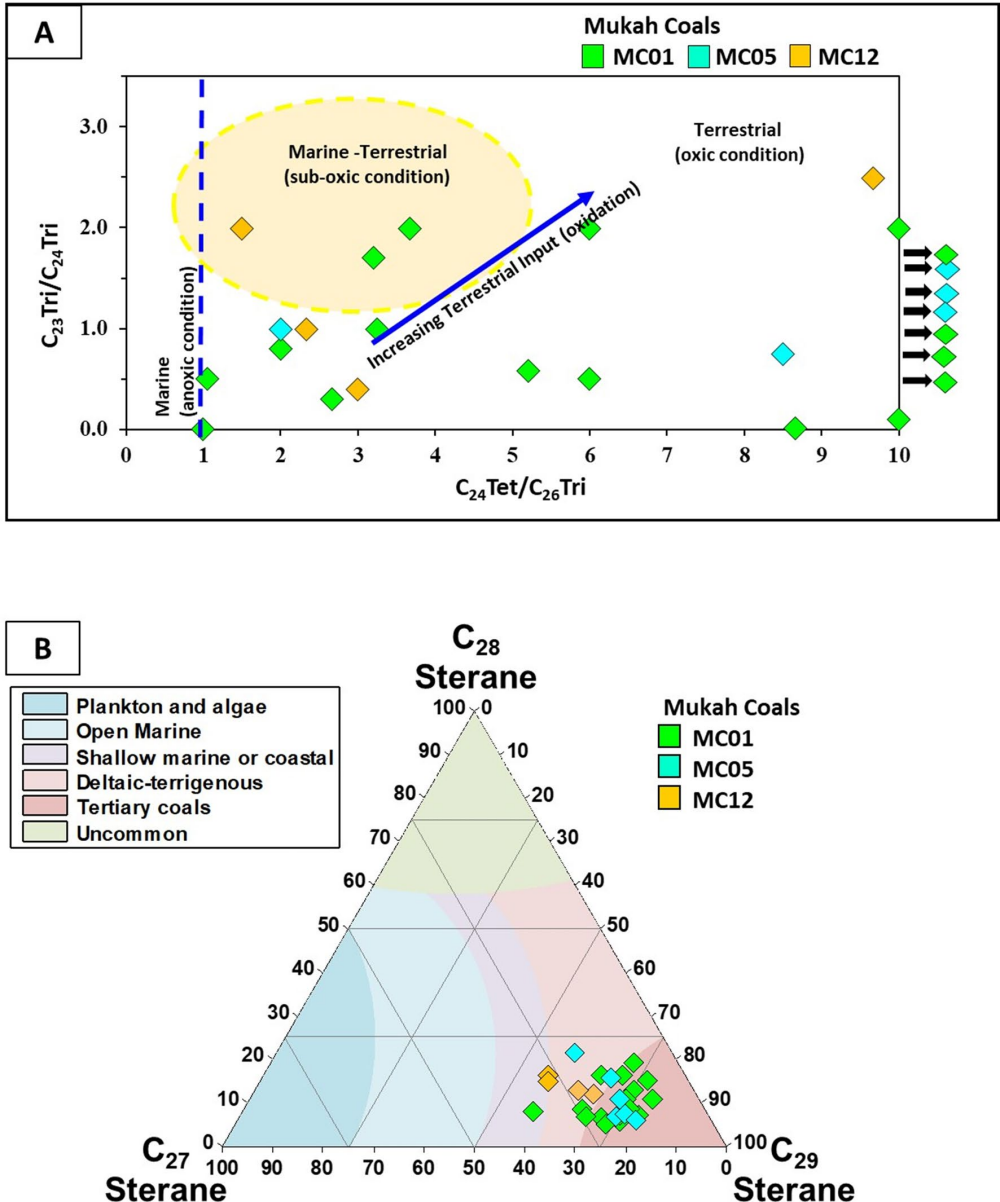
Plots of (**A**) C_23_Tricyclic/C_24_Tricyclic versus C_24_Tetracyclic/C_26_Tricyclic, and (**B**) C_27_, C_28_, and C_29_ of regular steranes, showing the organic matter source input for the studied Mukah coals, Sarawak.

Additionally, the Pr/Ph and Pr/*n*-C_17_ ratios can be combined to distinguish between marine and terrestrial OM inputs (e.g.^9, 43, 44, 50^); whereby, marine OM has Pr/Ph ratios < 1.5 and Pr/*n*-C_17_ ratios < 0.5^51, 52^, and the increasing contribution of terrestrial OM source will lead to higher ratios^53^. The relationship between the Pr/Ph and Pr/*n*-C_17_ ratios in this study suggests a mixed organic matter source with a large volume of terrestrial OM inputs (Fig. 13b). This inference is also in line with the high values of 4β (H)-Eudesmane sesquiterpane and C_29_/C_30_ hopane, indicating a precise marker of higher plant origin from β-Eudesmol constituent^54^ and a typical source of terrestrial organic matter^24^, respectively. The presence of large amounts of plant organic matter are also suggested from the relatively high proportions of Tm compared to Ts in the aliphatic hydrocarbon fraction of the analyzed coal samples (Fig. 9a), as the relative abundance of Tm is indicative for organic matter derived from land plants^25, 49^. This is compatible with the distribution of the tricyclic terpanes and their ratios, which are typical of aquatic-derived organic matter^55–58^. In this respect, the dominance of tetracyclic over tricyclic (Fig. 9a) suggests high amounts of accumulated terrestrial organic matter during the deposition of the coal sediments. Additionally, the combination of the C_24_Te/C_26_T and C_23_/C_24_ tricyclic terpanes ratios further provides a good indication for large amounts of terrestrial organic matter input deposited under suboxic to oxic environmental conditions (Fig. 14a).

The large amounts of higher plant input in the investigated coal samples are further highlighted by the homologous series of C_27_-C_29_ regular sterane in the *m/z* 217 ion fragmentogram (Fig. 9b). The abundance of C_29_ regular steranes of more than 50% in the saturated hydrocarbon fraction (Table 3) is normally suggestive of the presence of Tertiary coals containing mainly terrestrial OM inputs as demonstrated from a ternary diagram developed by^59^ (Fig. 14b). A standard ratio of C_29_/C_27_ steranes provides values of generally > 1 (Table 3), further implying that the Mukah coals were primarily terrigenous.

**Figure 15:**
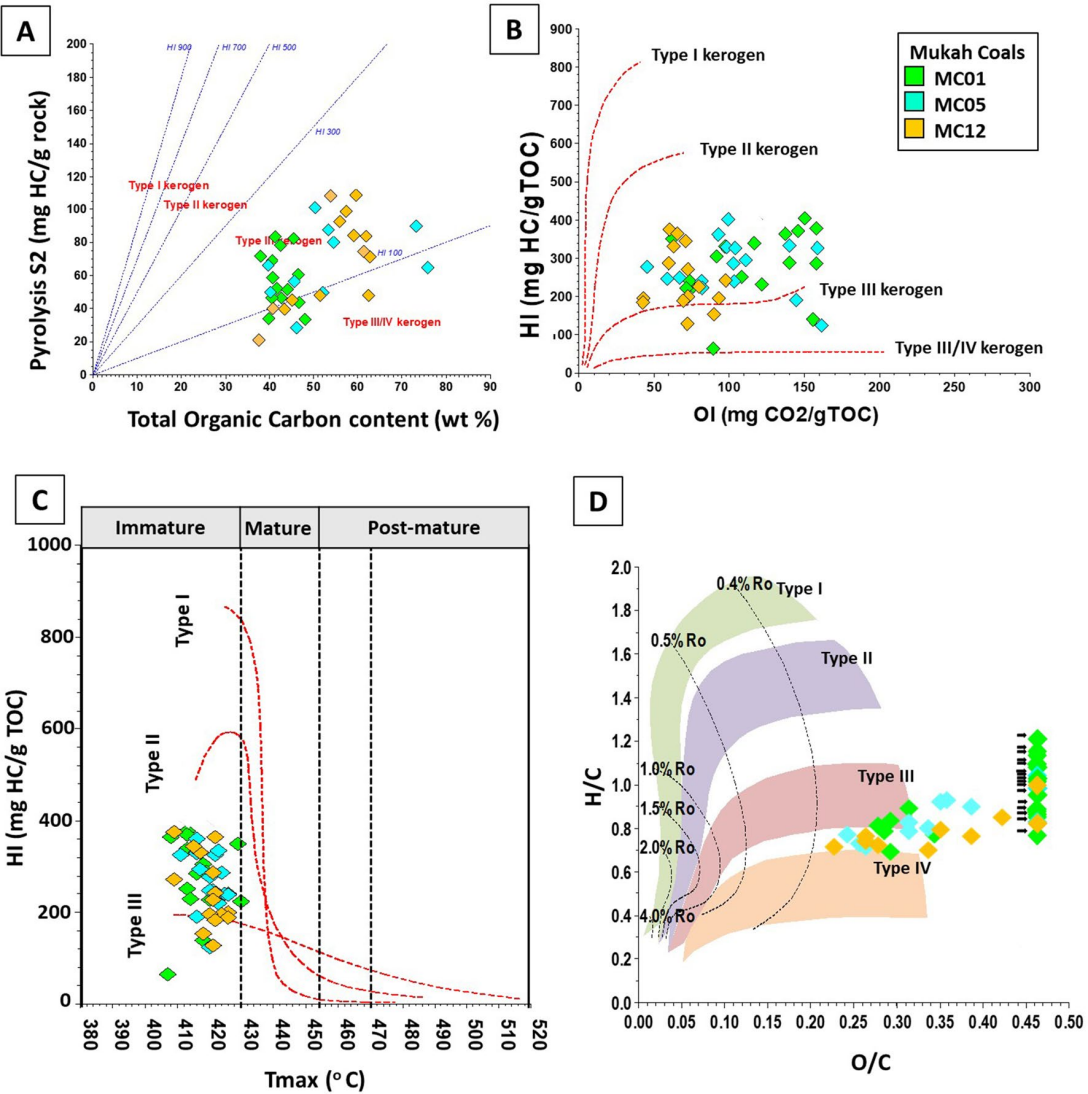
Plots of the analysed coal samples shows distribution data of (**A**) pyrolysis *S*_2_ yields versus total organic carbon content (TOC); (**B**) hydrogen index (HI) versus oxygen index (OI), (**C**) hydrogen index (HI) versus T_max_ (°C), and (**D**) atomic ratios of H/C versus O/C ratios (modified after Van Krevelen, 1961), representing the kerogen types of the Mukah coals, Sarawak.

The large contribution of terrestrial plant input is compatible with the geochemical data. As shown in the HI versus OI plot of the studied samples, the Mukah coals were classified as Type III kerogens and predominantly mixed Type II/III (Fig. 15b), which is in accordance with the HI versus T_max_ plot (Fig. 15c). The samples that contained HI values lower than 200 mg HC/mg rock are consistent with Type III kerogens derived from higher plants; whereas, the samples with HI values of more than 200 mg HC/mg rock are typical of mixed Type II/III kerogens which would be expected to receive mixed organic matter. This is in good agreement with the distribution of organic matter carbon content and hydrocarbon yield (Fig. 15a), and the plot of H/C and O/C atomic ratios (Fig. 13d), mainly indicating a terrestrial source for the analysed coals^60^. In addition to this, the ratio of C/N is reported to be greater than 20 and 40 for the investigated samples, further representing the organic input from higher plants^61^.

It has been found that almost all OM contain pyrite which is hard to be removed^62^. The studied coals have an atomic S/C ratio ranging from 0.00 to 0.02, typical of low sulfur Types II/III and Type III, based on the analyses and observations reported in^62, 63^. The work by^63^ concluded that atomic S/C ratios greater than 0.04 was assigned as Type II-S kerogens for the Miocene onshore-offshore, Santa Maria Basin, California; whereas^62^ has identified Type I-S kerogen with an atomic ratio of 0.057 from the Cambrian Tarim Basin, China. The low sulfur content and unfavorable conditions for pyrite formation in this study are attributed to the Mukah coal’s domed peat growth and corroborated with freshwater type coal facies that were further discussed in^11^.

### Peat-forming vegetation

References of peat-forming vegetation were made to the known ecological data of ancient peats in Malaysia and Brunei indicated by^64–68^. The vertical profile (Fig. 5) appears to be predominated by the freshwater peat swamp communities. Meanwhile, the riparian, strand forest, ferns, and mangrove swamp vegetation, which are present in low frequencies, support the ecology within the main lowland forest. The rich preservation of angiosperm pollen suggests that mostly terrigenous inputs are fed into the organic matter in dense and lowland forest vegetation. Overall, the plant communities appear to be represented mainly within freshwater-influenced swamps. The presence of *Calamus* type, *Barringtonia*, *Palaquium*, *Pandanus*, and *Stenochlaena palustris* in a few studied coals represent a former riparian fringe type of forest^66, 67^. The occurrence of mangrove pollens in sections MC12 and MC01 in small quantities suggests the mangrove pollens were within proximity to the peat swamp forest communities. In contrast, no indication of mangrove influence is recorded in section MC05. Although pteridophytic spores of monolete smooth and *Stenochlaena palustris* are found in the throughout the studied coals, they are not abundant, suggesting that the plant types were slightly influenced by fern taxa^66, 67^. Moreover, the significant occurrence of *Casuarina* type in the middle part of section MC12 and the small amount of pollen in most of the samples suggests that the Mukah coals were deposited close to the sea under the influence of the strand forest^66, 67^.

The assemblage list recovered in this study is similar to that provided by^1^. Compared with their work, this study yielded approximately similar assemblages of palynomorphs, but in slightly different proportions. The overwhelming presence of the *Casuarina* and *Calamus* types in the Mukah coals further suggests that the peat swamp vegetation was closely linked to the Kerapah/Kerangas peat forest and marginally bordered by rattan^1^ even though these pollens were found restricted to certain seam intervals of the studied coals.

The source of vegetation for the Mukah coals is in a good agreement with the biomarker distributions. The occurrences of aliphatic diterpanes such as *ent*-beyerane, isopimarane, phyllocaldanes, and kaurene type compounds have been found in most of the studied samples, with *ent*-beyerane and isopimarane being the predominant diterpanes (Fig. 8c). *Ent*-beyerane originates from *ent*-beyerene^69, 70^, while isopimaric acid is the precursor of isopimarane^71^. These diterpanes can also derived from labdane derived copalyl pyrophosphate^72^. According to^73^, plant speciation can be deduced from the relationship between plants and diterpene compounds as follows: (1) pimaranes are present in gymnosperms and pre-gymnosperms (cordaites) for Carboniferous, angiosperms, pteridophytes and bryophytes; (2) *ent*-beyerane in gymnosperms, pre-gymnosperms, and angiosperms; (3) kaurane in angiosperms, gymnosperms, pre-gymnosperms, bryophytes; and (4) phyllocladanes in gymnosperms and pre-gymnosperms. The presence of these diterpanes indicates the plant speciation are derived mainly from angiosperms as confirmed by the palynomorph assemblages. The presence of tetracyclic terpanes such as De-A-olean-13(18)-ene, De-A-Lupane and De-A-olean-12-ene in the studied Mukah coals, furthermore, confirms the dominance of angiosperms input into the organic matter which are derived from the oxygenated triterpenoids compounds like α amyrin, β amyrin, and lupeol during diagenesis of angiosperms^74–76^. Additionally, this present study has identified the incidence of plant-derived pentacyclic triterpenoids oleanane, indicating the existence of angiosperms as the major source of organic matter in the paleomires (e.g.^77, 78^).

### Controls on peat formation

Following the coal-forming models^79–87^, the authors inferred that different characteristics, architectures, and thicknesses of coal seams are governed by the height of the mire water table, which can be correlated with transgressive–regressive cycles, hence, may reflect different system tracts and the significance of key sequence-stratigraphic surface development in coal-bearing strata. Since the Mukah coals were deposited in a low-lying coastal setting, the groundwater and seawater are hydrologically connected. Thus, the rise in relative sea level can cause the groundwater table to rise in coastal mires, resulting in accommodation space for peat accumulation and upstream deposition of siliciclastics in the study area as the accumulation of peat requires a balance between the rate of accommodation and peat production (AR/PPR)^79–81^.

Overall, the Mukah coal formation initially occurred at a low water table that allowed the peat to form in peat-forming mires. The peat growth progressed within an overall rising water table (base level). During the peat formation, the creation of accommodation space was moderate to high in response to the rate of change in the base level. A gradual decrease in the rate is expected at the end of peat growth, which allowed the peat to accumulate and maintain a steady pace with the rise in the water table (Fig. 11). In some cases, particularly in the upper section of the Mukah coals, coal formation occurred in the mires during a high-water table, which resulted in high peat accumulation, followed by stable water table conditions, and balanced peat accumulation (Fig. 11). Since the Mukah coals were located in a coastal setting, peat accumulation and preservation were, therefore, associated with the sea-level rise in the paleo-mires, as indicated by the balanced to high AR/PPR. The balance in AR/PPR may cause partial exposure of the paleo-peat bodies in the mires; whereas, high AR/PPR may cause the drowning and inundations of paleo-peat bodies. Therefore, it is suggested that the proposed vertical variations in the lithotypes, microlithotypes, and macerals have been controlled by fluctuations in the groundwater level in ancient freshwater mires, thereby resulting in different accommodation/peat preservation rates (Fig. 11). High huminite, low inertinite, and high humite and clarite contents in most of the studied coals indicate permanently water-saturated peat with balanced to high accommodation creation^82^. The increased liptinite content in the middle section of the Mukah coals indicates a loss of biomass and poor preservation of woody tissues. This may reflect the high preservation of herbaceous vegetation (unstructured material) in the mires. The moderate detrital-mineral matter content in topogenous peats may imply the association of fluvial input with the mires when peat accumulation was unable to keep up with the high rates of accommodation.

In this study, a large number of thin and clean coal seams overlain by tidal flat deposits of Begrih Formation^8^ indicate that frequent changes in the peat-forming mires occurred during the accumulation of peat, as evidenced by the temporary development of paleo-peat bodies from ombrotrophic to mesotrophic to rheotrophic and vice versa (Fig. 11). Based on the compositional variations of macerals, sub-macerals, and detrital-mineral matter, these changes were controlled by the moderate to high eustatic rise in sea level that led to a continuous rise in the base level of the region and subsequent rise in the water table of the coastal plain. The variations of coal lithotypes from being coarsely banded to dull in nature support intermittent moderate to high flooding of the fluvial areas, resulting in moderate to high diversification of the macerals/submacerals and moderate to no amount of mineral matter (Fig. 11). Once the mires were formed, they were shielded from substantial clastic deposition, as shown by the moderate to low amounts of clastic material in the seams (Fig. 11). On the basis of these observations, it is suggested that the anticipated stability of the tectonic setting should be excluded during the development of peat. This is supported by the study conducted by^88^, which showed that shifts in the base-level from Middle Miocene to Pliocene were highly influenced by eustasy; whereas, during Late Oligocene to Early Miocene, the effects of global sea-level changes were significantly obscured by major tectonic movements. Moreover, the syn-collisional event between Luconia Block–Dangerous Grounds and Borneo in a rapidly subsiding basin that influenced the formation of the Early Miocene Mukah coals^2^ may have greater significance locally within the depositional basin in controlling the properties of coal seams (i.e., thickness, composition, continuity, and geometry)^89^, and is comparable according to structural characteristics and overall subsidence that occurred on land within the Sarawak basin^90^.

The water table fluctuations in the study, however, are strongly dependent on climate changes, as the influence of relative sea level on the position of regional water tables diminishes further inland^90–92^. The compositional variations of coal lithotypes in the Mukah coals have most likely been affected by climatic conditions as the changes may be attributed to the relative sea-level variables of the region. In this study, the obvious absence of major fire-generated inertinite coal seams, uncommon dull coal, and dulling-upward succession from the base to the top of the coal seams could be related to the ever-wet climate during the Neogene, as the studied coals may have experienced a widespread rise in rainfall^93–96^, which corresponded to the Asian monsoon from the earliest Miocene onward. As previously reported, the structured huminite maceral content (humotelinite) is predominant in the Mukah coals; thus, this observation supports permanently water-saturated mires with a minimum oxidation level during peat accumulation and was influenced by the rise in the water table. Furthermore, the high content of humite-clarite throughout the coals implies that the paleomires have experienced a prolonged rise in sea level, as the signatures are typical of “transgressive” coals^87^. Based on the petrographic evidence, the Mukah coal peat mires evolved on a wet, low-relief coastal plain in low-lying areas with continuously intermittent moderate to high flooding. The relationship between low inertinite (< 10 vol%), moderate to high diterpanes ratio (R_dit_) (*m/z* 123), palynology signatures, and low inertinite–vitrinite (IV) factor suggests that a moderate to high-water table had influenced the development of peat in the wet mires while the climate was probably more wet during these periods. The scarcity of gymnospermous pollen (*Podocorpus*) in the coals may further indicate a low contribution from vegetation in the drier or upland areas. This is further supported by^1^, who reported the abundance of *Casuarina*-type pollen associated with common occurrences of *Dacrydium* in the Kerapah peat swamps, indicating an extremely wet climate. The presence of *Casuarina* type in high frequency is also the case of this study.

Peat accumulation rates vary considerably, particularly with respect to geographical latitudes^84^. It is proposed in this study that the average peat accumulation rate for low-lying Holocene peats at low latitudes (< 10°) ranges from ~ 2 to ~ 5 mm/year. According to^97^, the compaction ratio to form low-rank coal seams from their original peats varies between 1.4:1 and 30:1. A ratio of 10:1 has been most commonly used^98–100^. Considering that the coals being studied are low in rank and accumulated in a tropical climate, a ratio of 10:1 and peat accumulation rates of 2–5 mm/year have been used as conservative estimations, resulting in the original thickness of the peat deposits forming the Mukah coals of the Balingian Formation to be between 5 and 35 m, while the peat-forming environments were established between 10,000 and 175,000 years ago. The calculation is strongly dependent on the assumed peat–coal ratio; thus, it shows that the deposition of peat for the Mukah coals occurred within a short time interval.

### Mukah coals in a sequence-stratigraphic context

Several key sequence-stratigraphic surfaces have been proposed in the studied paralic coal beds of the Balingian Formation (Fig. 11). The coal that initially formed at a low water table, overlies the paludification surface (PaS); meanwhile, the gradual termination of the peat represents a give-up transgressive surface (GUTS) in response to an increasing accommodation rate. Furthermore, the coal bed overlies a terrestrialization surface (TeS) at its initiation of peat formation in response to a decreasing accommodation rate, resulting in a GUTS during a stable water table. An accommodation reversal surface (ARS) was identified during the transition of accommodation from decreasing to increasing (balanced to high AR/PPR) or increasing to decreasing (high to balanced AR/PPR).

**Figure 16:**
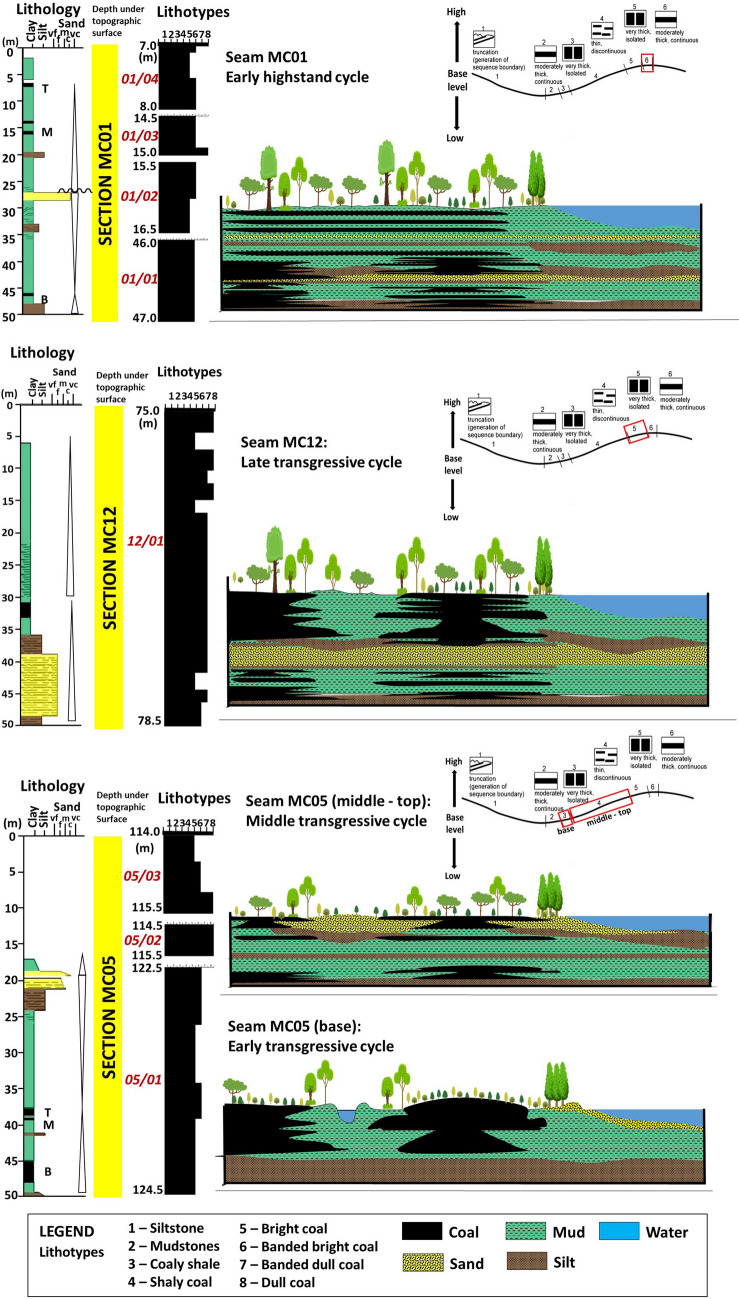
Construction of the accumulation progress during different stages in the evolution of the analyzed Mukah coal seams, Balingian Formation, Sarawak.

In this study, the onset and amount of peat accumulation is observed from the difference in the thickness of the coals, which could be linked to the period of the base level changes in the mires. A shorter period of rising base level is assumed to have occurred in the middle and top parts of the section. The lowest coal seam (05/01) within the lower stratal section (MC05) is thicker than the upper and middle coal seam, suggesting that the initial transgressive stage occurred during the formation of the peat^79^, as the increasing base level increases the rate of accommodation, resulting in the formation of a moderately thick coal bed (Fig. 16). Meanwhile, the development of the middle (05/02) and upper (05/03) coal seams in section MC05 is suggested to occur during the middle transgressive stage (Fig. 16), as their thicknesses are relatively thin and isolated owing to the high accommodation rate during peat accumulation in the mires^79^. The increase in the rate of change in the base level is supported by the transition to a coarsening-upward unit, which indicates the gradual decrease in the growth of the peat-forming environment into a more clay- and sand-rich deposition (Fig. 16). The greater thickness of the middle section coal at MC12 indicates an overall increase in base level with a moderate water table in the initial stage, and that a late transgressive stage (Fig. 16) may have influenced the formation of peat in the mires^79^. From the associated sedimentary deposits within the coals, the overall fining upward succession further supports an upward decrease in depositional energy in the mires, thereby decreasing the rate of change in the base level. The occurrences of thin coal seams in the upper section (MC01) indicate that shifts in the base level have occurred over a relatively short period under stable peat-forming conditions (Fig. 16). The continuous formation of thin coal seams within the upper stratal section may suggest that the initial highstand stage (Fig. 16) controlled the low to moderate accommodation rate during the accumulation of peat in the mires^79^. The overall fining upward cycle indicates that hydraulic energy is low during deposition, thereby supporting the interpretation of gradual overgrowth peat in the mires.

In this study, the overall rising water table level during the growth of paleo-peat bodies suggests that the Mukah coals were deposited in a fluvial system. In the sequence-stratigraphic framework, the architecture of the Mukah coals from the lower to upper section could represent the transition from transgressive (TST) to initial highstand (HST) cycles (Fig. 16). This interpretation is supported by the high content of humite/vitrite + clarite throughout the studied coals (Fig. 16), which corresponds to a transgressive event influencing the growth of peat in the mires^100^. The interpretation may also be linked to Sarawak’s stratigraphic framework analysis^101^, the detailed offshore reservoir geological assessment in the Balingian province^101, 102^, and the paleogeographic study of the Balingian Formation^8^, which suggest that shales and coals of offshore Cycles I–II were deposited in an overall transgressive environment^101–103^, while two deposition stages, comprising an early transgressive event, followed by a late regressive episode, influenced the onshore Balingian Formation^8^.

### Data and methods

A total of 45 coal samples were collected from eight coal seams encountered in three mining boreholes (MC01, MC05, and MC12) in the Mukah coalfield, Balingian Formation, with bed thicknesses ranging from 1 to 3.5 m (Fig. 2). Prior to sample selection, a detailed analysis of the lithologic and coal lithotypes from the base to the top of the seams was performed in high-resolution observation based on the brightness system indicated by^104,105^.

Organic petrographic analysis was performed under a plane-polarized reflected light using a Leica DM 6000 M microscope and Leica CTR6000 photometry system equipped with fluorescence illuminators. Huminite/vitrinite reflectance measurements were performed in reflected white light under oil immersion with a refractive index (ne) of 1.518 at 23 °C. Calibration was performed with a Leuco Sapphire standard with a 0.589% reflectance value. Reported vitrinite reflectances (%Ro) are the averages of 100 measurements per sample and mean random vitrinite reflectance values were calculated using Hilgers Diskus Fossil software. Coal rank determination and maceral classification were based on the technique discussed by^106^. Maceral and combined maceral-microlithotype analyses were used to determine the petrographic composition of polished blocks, as described by^107^. Polished blocks were prepared following the work described by the International Committee for Coal and Organic Petrology^108^. At least 1000-point counts of both analyses were performed in which the identification process adapted the terminology for brown coals developed by ICCP for huminite^109^, liptinite^110^, and inertinite^111^ nomenclature. Fluorescence was used for liptinitic maceral identification. Samples with a high sporinite percentage were used for palynological investigation. The analysis procedure and identification of 100–200 counts of palynomorphs on each slide were recorded, following the standards^112^.

Organic geochemistry was performed on the samples’ total organic carbon content (TOC, wt%) using a Multi N/C 3100 analyzer, and element concentrations of carbon (C), hydrogen (H), nitrogen (N), sulfur (S), and oxygen (O) were studied using an Elementar Analysensysteme GmbH (vario MICRO cube) analyzer. From the total organic carbon content data, samples were screened, and the selected samples were sent for Rock–Eval pyrolysis analysis to assess the source richness, kerogen type, hydrocarbon potential, and maturity of the studied coals. The samples were crushed to < 150 mm and analysed using Rock–Eval Pyrolysis 6 as described by^113^. Selected samples were analyzed for bitumen extraction and biomarker analysis. Source rock characteristic assessments using TOC, bitumen, and hydrocarbon yields are based on the work by^25, 26^. The aliphatic fractions of coal extracts were collected for gas chromatography and gas chromatography–mass spectrometry. Biomarker traces were identified based on their elution pattern, retention time, and by comparing published works by^24–26, 29, 33, 114–117^.

## Conclusion

The following conclusions can be drawn from the above:

1. The Mukah coals are defined as lignite to sub-bituminous B types, characterized by high huminite and clarite. Little or no sulfur and pyrite were observed. The vertical changes in coal-forming vegetation and temporary development of rheotrophic–ombrotrophic mires, as represented by the changing petrographic compositional variation, may have occurred because of the water table fluctuations, which resulted in various ecological groups of plants.
2. The source of the Mukah coals is suggested to have been derived mainly from arborous vegetation, which appears to be predominated by the freshwater peat swamp communities.
3. Rapid subsidence and an ever-wet climate may be attributed to the relative sea-level variation in the region. This may have resulted in water table fluctuations, balanced to high peat accumulation and preservation ratios during the peat formation in mires, moderate diversification of the composition in the coals, as well as a large number of thin and clean coal seams, characterized by coarsely banded to dull coal lithotypes with moderate mineral matter and low inertinite.
4. Two patterns of key sequence-stratigraphic surfaces are mainly proposed in this study: PaS–ARS–GUTS, in response to the increasing accommodation rate (balanced to high AR/PPR); and TeS–ARS–GUTS, in response to the decreasing accommodation rate (high to balanced AR/PPR) during periods of a stable water table.
5. Within the fluvial setting, peat formation in the mires could be influenced by TST to initial HST cycles. The Mukah coals may correspond to peat deposits with a minimum thickness of ~ 5 m, which are expected to have formed between 10,000 and 175,000 years ago.

## Supplementary Information

Supplementary Information.
