# Supplementary material for: Paleoenvironment reconstruction and peat-forming conditions of Neogene paralic coal sequences from Mukah, Sarawak, Malaysia
Source: Sci Rep. 2022 May 25;12:8870. doi: 10.1038/s41598-022-12668-6 (PMC9132988; doi:10.1038/s41598-022-12668-6)
Supplement: Supplementary file 1 — Supplementary Information. [file 41598_2022_12668_MOESM1_ESM.docx]

# Appendix I

Peak assignments for hydrocarbons in chromatograms of saturated fractions in the *m/z* 123 (I), *m/z* 191 (II) and *m/z* 217 (III) mass fragmentograms (use for reference to explain Fig. 8-9).

| (I) Peak *no*. | Compound abbreviation | C_no_ |
| --- | --- | --- |
| Sesquiterpanes |  |  |
| s1 | C_14_ Bicyclic sesquiterpane |  |
| s2 | C_14_ Bicyclic sesquiterpane |  |
| s3 | C_14_ Nordrimane |  |
| s4 | C_15_ Bicyclic sesquiterpane |  |
| s5 | 4β Eudesmane |  |
| s6 | 8β(H)-Drimane (C_15_) |  |
| s7 | 8β(H)-Homodrimane (C_16_) |  |
| s8 | C_16_ Bicyclic sesquiterpane |  |
|  |  |  |
| Diterpanes |  |  |
| d1 | C_18_ Diterpane |  |
| d2 | Labdane |  |
| d3 | 19-Norisopimarane |  |
| d4 | Demethylated ent-beyerane? |  |
| d5 | Sandaracopimarane |  |
| d6 | ent-Beyerane |  |
| d7 | iso-Pimarane |  |
| d8 | Demethylated ent-beyerane? |  |
| d9 | 16β-Phyllocladane |  |
| d10 | 16α-Kaurane |  |
| d11 | 16α-Phyllocladane |  |
| d12 | 16β-Kaurane |  |
|  |  |  |
| Rdit = (2 + 4 + 6)/(3 + 5 + 7) | |  |
| (II) Peak *no*. | Compound abbreviation |  |
| Tetracyclic Terpanes |  |  |
| 6 | De-A-olean-13(18)-ene |  |
| 7 | De-A-Lupane |  |
| 8 | De-A-olean-12-ene |  |
| 9 | 17,21-Secohopane (C25) |  |
|  |  |  |
| Pentacyclic Terpanes |  |  |
| 10 | 17α(H),18α(H),21β(H)-28,30-Bisnorhopane |  |
| 11 | 17α(H), 21β(H)-25-Norhopane |  |
| 12 | 18α(H),22,29,30-trisnorneohopane | Ts |
| 13 | 17α(H),22,29,30-trisnorhopane | Tm |
| 14 | 28,30-Bisnorhop-13(18)-ene |  |
| 15 | Oleana-2,13(18)-diene 24 |  |
| 16 | Oleana-2,12-diene |  |
| 17 | 17α,21β(H)-nor-hopane | C_29_ hop |
| 18 | Olean-12-ene + Ursa-2,12-diene |  |
| 19 | Hop-17(21)-ene |  |
| 20 | 17α,21β(H)-hopane | Hopane |
| 21 | Hop-17(21)-ene |  |
| 22 | 17 β,21α (H)-Moretane | C_30_Mor |
| 23 | αβ-Hopane |  |
| 24 | Neohop-13(18)-ene |  |
| 25 | ββ-30-Norhopane |  |
| 26 | 17α,21β(H)-homohopane (22S) | C_31_(22S) |
| 27 | 17α,21β(H)-homohopane (22R) | C_31_(22R) |
| 28 | ββ-hopane |  |
| 29 | 17α,21β(H)-homohopane (22S) | C_32_(22S) |
| 30 | 17α,21β(H)-homohopane (22R) | C_32_(22R) |
| 31 | 17α,21β(H)-homohopane (22S) | C_33_(22S) |
| 32 | 17α,21β(H)-homohopane (22R) | C_33_(22R) |
| (III) Peak *no*. | Compound abbreviation |  |
| a | 13β,17α(H)-diasteranes 20S | Diasteranes |
| b | 13β,17α(H)-diasteranes 20R | Diasteranes |
| c | 13α,17β(H)-diasteranes 20S | Diasteranes |
| d | 13α,17β(H)-diasteranes 20R | Diasteranes |
| e | 5α,14α(H), 17α(H)-steranes 20S | ααα20S |
| f | 5α,14β(H), 17β(H)-steranes 20R | αββ20R |
| g | 5α,14β(H), 17β(H)-steranes 20S | αββ20S |
| h | 5α,14α(H), 17α(H)-steranes 20R | ααα20R |
